# Lifestyle and Horizontal Gene Transfer-Mediated Evolution of *Mucispirillum schaedleri*, a Core Member of the Murine Gut Microbiota

**DOI:** 10.1128/mSystems.00171-16

**Published:** 2017-01-31

**Authors:** Alexander Loy, Carina Pfann, Michaela Steinberger, Buck Hanson, Simone Herp, Sandrine Brugiroux, João Carlos Gomes Neto, Mark V. Boekschoten, Clarissa Schwab, Tim Urich, Amanda E. Ramer-Tait, Thomas Rattei, Bärbel Stecher, David Berry

**Affiliations:** a Division of Microbial Ecology, Department of Microbiology and Ecosystem Science, University of Vienna, Austria; b Max von Pettenkofer Institute of Hygiene and Medical Microbiology, Ludwig Maximilians University of Munich, and German Center for Infection Research (DZIF), Partner Site LMU Munich, Munich, Germany; c Food Science and Technology Department, University of Nebraska—Lincoln, Lincoln, Nebraska, USA; d Nutrition, Metabolism, and Genomics Group, Wageningen University, Wageningen, the Netherlands; e Archaea Biology and Ecogenomics Division, Department of Ecogenomics and Systems Biology, University of Vienna, Vienna, Austria; f Division of Computational Systems Biology, Department of Microbiology and Ecosystem Science, University of Vienna, Vienna, Austria; University of Colorado, Denver

**Keywords:** DNRA, *Deferribacteres*, gut microbiota, *Helicobacter*, fluorescence *in situ* hybridization, metatranscriptomics

## Abstract

*Mucispirillum schaedleri* is an abundant inhabitant of the intestinal mucus layer of rodents and other animals and has been suggested to be a pathobiont, a commensal that plays a role in disease. In order to gain insights into its lifestyle, we analyzed the genome and transcriptome of *M. schaedleri* ASF 457 and performed physiological experiments to test traits predicted by its genome. Although described as a mucus inhabitant, *M. schaedleri* has limited capacity for degrading host-derived mucosal glycans and other complex polysaccharides. Additionally, *M. schaedleri* reduces nitrate and expresses systems for scavenging oxygen and reactive oxygen species *in vivo*, which may account for its localization close to the mucosal tissue and expansion during inflammation. Also of note, *M. schaedleri* harbors a type VI secretion system and putative effector proteins and can modify gene expression in mucosal tissue, suggesting intimate interactions with its host and a possible role in inflammation. The *M. schaedleri* genome has been shaped by extensive horizontal gene transfer, primarily from intestinal *Epsilon*- and *Deltaproteobacteria*, indicating that horizontal gene transfer has played a key role in defining its niche in the gut ecosystem.

**IMPORTANCE** Shifts in gut microbiota composition have been associated with intestinal inflammation, but it remains unclear whether inflammation-associated bacteria are commensal or detrimental to their host. Here, we studied the lifestyle of the gut bacterium *Mucispirillum schaedleri*, which is associated with inflammation in widely used mouse models. We found that *M. schaedleri* has specialized systems to handle oxidative stress during inflammation. Additionally, it expresses secretion systems and effector proteins and can modify the mucosal gene expression of its host. This suggests that *M. schaedleri* undergoes intimate interactions with its host and may play a role in inflammation. The insights presented here aid our understanding of how commensal gut bacteria may be involved in altering susceptibility to disease.

## Abstract (precis)

Shifts in gut microbiota composition have been associated with intestinal inflammation, but it remains unclear whether inflammation-associated bacteria are commensal or detrimental to their host. Here, we studied the lifestyle of the gut bacterium *Mucispirillum schaedleri*, which is associated with inflammation in widely used mouse models. We found that *M. schaedleri* has specialized systems to handle oxidative stress during inflammation. Additionally, it expresses secretion systems and effector proteins and can modify the mucosal gene expression of its host. This suggests that *M. schaedleri* undergoes intimate interactions with its host and may play a role in inflammation. The insights presented here aid our understanding of how commensal gut bacteria may be involved in altering susceptibility to disease.

## INTRODUCTION

**FIG 1:**
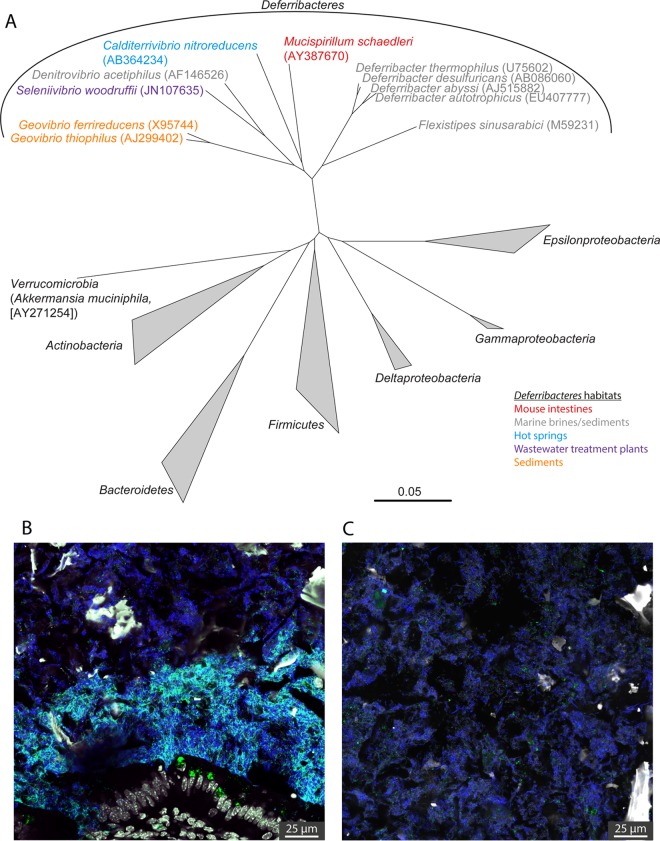
Phylogeny and habitat of *Mucispirillum*. (A) Phylogenetic tree of *M. schaedleri* in relation to other cultured members of the *Deferribacteres* and abundant gut taxa based on the maximum-likelihood method using the 16S rRNA gene and 500 bootstraps. The source of isolation of the members of the *Deferribacteres* is indicated by color. GenBank accession numbers are shown in parentheses. Scale bar, 0.05 change per nucleotide position. (B and C) Fluorescence *in situ* hybridization image of the ceca of ASF^4^ mice colonized with *M. schaedleri* ASF 457 (Cy3, green), showing localization proximal to the mucosa (B) and its almost complete absence in the lumen (C). All bacteria (targeted by the EUB338I-III mix, Cy5) are blue, and DAPI (4′,6-diamidino-2-phenylindole)-stained cells are shown in gray.

*Mucispirillum schaedleri* is a non-spore-forming, flagellated anaerobe with a spiral or broken-stick morphology thought to assist movement through the viscous gut mucus layer (1, 2). *M. schaedleri* has a long history of being included in defined microbial consortia for gnotobiotic laboratory animal studies (3–5) and is one of eight species in the widely used category altered Schaedler flora (ASF) (6). Members of the genus *Mucispirillum* has been detected in a variety of hosts, including pigs, goats, dogs, rats, mice, turkeys, termites, and cockroaches (7–15). *M. schaedleri* is a core member of the laboratory mouse microbiota and can colonize the intestinal tract from the stomach to the colon (16). As part of the phylum *Deferribacteres* (17) (Fig. 1), *Mucispirillum* stands out as one of the few taxa (genus classification and above) commonly found in mice but not humans (18). It has, however, occasionally been detected in humans (19), which may be due to either transient or infrequent colonization or its presence at an abundance below the detection limit of standard sequencing efforts.

*Mucispirillum* has been associated with both inflammatory markers and active colitis in the T-bet^−/−^ Rag2^−/−^ mouse model, in chemically induced colitis, and during *Citrobacter rodentium* infection (20–23). ASF mice infected with *Helicobacter bilis* exhibited an IgG response to *M. schaedleri* (24), indicating that it can become the target of a systemic immune response potentially via translocation across the intestinal mucosal barrier (25). In a study of diet-induced weight modification, *Mucispirillum* was positively correlated with serum leptin levels (26), which may be a feed-forward loop to maintain its niche, as luminal leptin induces mucin secretion (27, 28). Leptin is also thought to be released into the lumen during colitis (29), which may contribute to *Mucispirillum* expansion during intestinal inflammation. Despite its localization to the mucus layer and association with mucus production, it has not, however, been identified as a significant degrader of host-derived compounds *in vivo* (30).

Though it is a core member of the murine gut microbiota and increases during conditions of inflammatory stress, the genetic and physiological features of *M. schaedleri* remain poorly understood. In this study, we analyzed and compared the draft genomes of two recently diverged lineages of *M. schaedleri* ASF 457. We performed physiological experiments to test key features predicted by the genomes. We also identified genes expressed by *M. schaedleri in vivo* using newly generated and previously published metatranscriptomic data from gnotobiotic and conventional mice. Together, these results provide a comprehensive picture of the evolution and intestinal lifestyle of this inflammation-associated mucus-dwelling bacterium and further our understanding of its potential to be an intestinal pathobiont.

## RESULTS

### Genomic features. (i) Genome reconstruction and comparison.

The assembled genomes of variants MCS and AYGZ have 36 and 39 contigs, respectively, and were estimated to be largely complete based on detection of a complete set of tRNAs and conserved housekeeping genes (see Table S1 in the supplemental material). The two genomes are very similar, with only a few shared genes having nonidentical sequences. The nonidentical, shared genes generally have high sequence identity (>99%) and include genes for hydrogenase 2, transposases, transporters, and multiple proteins with unknown functions, indicating that the genomes diverged little from one another and that differences consist of only a small number of single nucleotide polymorphisms.

10.1128/mSystems.00171-16.6 TABLE S1  General features of the genomes of *Mucispirillum schaedleri* ASF 457 substrains AYGZ and MCS. Download TABLE S1, PDF file, 0.3 MB. Copyright © 2017 Loy et al. 2017 Loy et al. This content is distributed under the terms of the Creative Commons Attribution 4.0 International license.

### (ii) Central metabolism.

**FIG 2:**
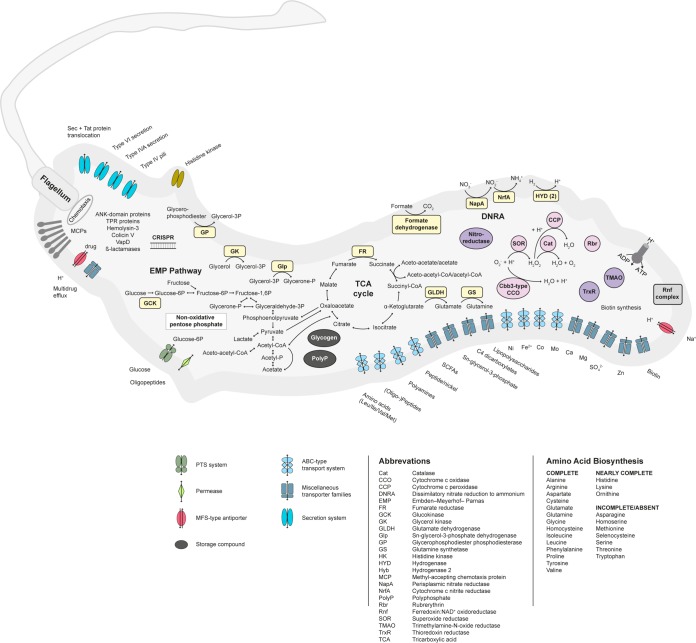
Selected genomic features of *M. schaedleri* ASF 457. Predicted metabolic and physiological capabilities based on genome annotations are shown.

The genomes predict that *M. schaedleri* altered Schaedler flora 457 (ASF 457) harbors a complete Embden-Meyerhof-Parnas (EMP) pathway and a nonoxidative pentose phosphate pathway, as well as a complete tricarboxylic acid (TCA) cycle that features a *Helicobacter*-type succinyl-coenzyme A (CoA):acetoacetate CoA transferase (SCOT; EC 2.8.3.8) (Fig. 2). It has complete biosynthesis pathways for most amino acids, but several pathways, such as for methionine and tryptophan, are incomplete or not detected (Text S1). *M. schaedleri* may therefore be reliant on amino acid or oligopeptide transporters for growth. Ammonia can be assimilated via a glutamate dehydrogenase (GdhA, EC 1.4.1.4), and a type 3 glutamine synthetase (GlnA, EC 6.3.1.2) (31). A description of cofactor and vitamin biosynthesis pathways, storage compounds, motility and chemotaxis genes, a clustered regularly interspaced short palindromic repeat (CRISPR) system, mobile genetic elements, and transporters can be found in Text S1.

10.1128/mSystems.00171-16.1 TEXT S1  Additional methods and results not included in the main text. Download TEXT S1, PDF file, 0.6 MB. Copyright © 2017 Loy et al. 2017 Loy et al. This content is distributed under the terms of the Creative Commons Attribution 4.0 International license.

### (iii) Putative electron donors and carbon sources.

The *M. schaedleri* genome features an extremely limited repertoire of polysaccharide degradation machinery, consisting of just 3 glycoside hydrolases (family 57 α-amylases) that are likely used for glycogen storage (Text S1). Genes for degradation of glycerophosphodiester and glycerol, as well as for transport of glucose, dicarboxylic acids, and short-chain fatty acids, were also detected (Text S1). The genomes encode 15 proteases and 3 aminopeptidases, a subset of which (4 for AYGZ, 5 for MCS) are predicted to be secreted. Catabolic pathways for glutamine, asparagine, and cysteine were identified. We detected multiple ABC transporters for amino acids, including for leucine, isoleucine, valine, and methionine. Transporters were also present for peptides and polyamines (ABC type), oligopeptides, and a permease for oligopeptides. We detected hydrogenase 2 (EC 1.12.7.2), which is a membrane-bound Ni-Fe hydrogenase that reduces menaquinone to menaquinol in a reversible reaction (32). Although we detected a formate dehydrogenase, which allows for reversible NAD-dependent interconversion of formate and CO_2_, we found neither hydrogenase 3 nor hydrogenase 4, so there is no evidence for the presence of a formate-hydrogen lyase.

### (iv) Respiration and oxidative-stress response.

*M. schaedleri* has genes for dissimilatory nitrate reduction to ammonia (DNRA), with a periplasmic nitrate reductase (NapA, EC 1.7.99.4) as well as a nitrite reductase (NrfA, EC 1.7.2.2). The presence of genes for fumarate reductase suggests that fumarate can also be used as a terminal electron acceptor for anaerobic respiration. We also detected genes for the membrane-bound Rnf complex, which is proposed to couple the electron transfer from reduced ferredoxin to NAD^+^ with the translocation of Na^+^ ions across the cytoplasmic membrane via a Na^+^-translocating ferredoxin:NAD^+^ oxidoreductase and thereby generate a sodium ion gradient (33).

*M. schaedleri* has genes for a high-affinity cbb3-type cytochrome *c* oxidase (EC 1.9.3.1), which may be used either for protection from O_2_ stress (34, 35) or for microaerobic respiration (36, 37). Several genes for detecting and defending against oxidative stress, including a superoxide reductase, catalase, cytochrome *c* peroxidase, rubrerythrin, and thioredoxin reductase, were detected. The genome also includes genes for a nitroreductase as well as other nitroreductase family proteins, which may be used for scavenging nitrogen radicals formed during nitrate and nitrite reduction. We detected genes for a putative trimethylamine-*N*-oxide reductase (EC 1.7.2.3) for the reduction of trimethylamine-*N*-oxide (TMAO) into trimethylamine (TMA), which may serve as a trophic link to methylotrophic methanogens in the gut that use methylated amino compounds like TMA in conjunction with H_2_ during methanogenesis (38).

### (v) Secretome and putative interaction genes.

Parts of either a type II secretion system (T2SS) or a type IV pilus biogenesis machinery (T4P) were present in the genome. The T2SS is widely distributed especially among *Proteobacteria*, most of which are extracellular pathogens, and is usually encoded by at least 12 genes in a single operon (39, 40). We detected genes for only 5 proteins, the major prepilins T2SC to T2SG. Seven of the T2SS core proteins (T2SH to T2SO) (41) seem to be missing, indicating either a nonfunctioning T2SS system or, alternatively, the presence of a T4P or DNA uptake machinery (42). A gene encoding PilZ, a putative T4P assembly protein, is located in close vicinity to the T2SS/T4P genes. We also detected a PilT protein, which has been proposed to be a force-generating protein for pilus retraction (43).

We detected a putative type IVA secretion system (T4ASS), including five Tra conjugal transfer proteins (TraC, TraD, TraF, TraG, TraL), three Trb conjugal transfer proteins (TrbB, TrbD, TrbG), and a VirB complex with a type IV secretion/conjugal transfer ATPase VirB4 family protein and a VirB8 family protein belonging to the putative T4ASS (44), which may mediate horizontal gene transfer (HGT) (45) via conjugation or play a role in pathogenicity (46, 47). Virulence-associated protein D (VapD) was found located between the Tra and Trb loci of the T4ASS and several transposases, integrases, and tRNA genes. The exact biological role of VapD has not yet been established, but it is known as alpha-toxin in *Haemophilus influenzae* and as a typical prokaryotic toxin with the activity of an mRNA interferase (48). Though many pathogens carry multiple *vap* genes, *Helicobacter pylori* also carries only the *vapD* gene (48).

The type VI secretion system (T6SS) consists of 13 conserved core proteins necessary for function (49). *M. schaedleri* has bacteriophage-like components of the T6SS, including hemolysin-coregulated protein (Hcp), valine-glycine repeat protein G (VgrG, putatively), TssB/C, which complexes to form a needle sheath (50), and TssE, which is homologous to the bacteriophage baseplate protein gp25 (51). It also has membrane-associated proteins TssL, TssM, TssJ, and other T6SS proteins with unknown functions (TssA, TssF, TssG, and TssK). Additionally, homologs to other associated proteins, such as ClpV and a putative eukaryote-like phospholipase D protein, which is involved in destabilization of the host cell membrane (52), were also detected.

Ankyrin repeats (ANK) are found primarily in eukaryotic genomes, but proteins with ANK domains are also present in some symbiotic and pathogenic bacteria (53). We detected a total of 10 genes with ANK domains in ASF 457. Eleven genes were identified with tetratricopeptide repeat (TPR) domains, which are involved in virulence in bacterial pathogens (54). In addition, we detected hemolysin-3, a putative colicin V production protein (55, 56), and seven genes encoding putative β-lactamases (EC 3.5.2.6).

### Putative horizontally transferred genes.

**FIG 3:**
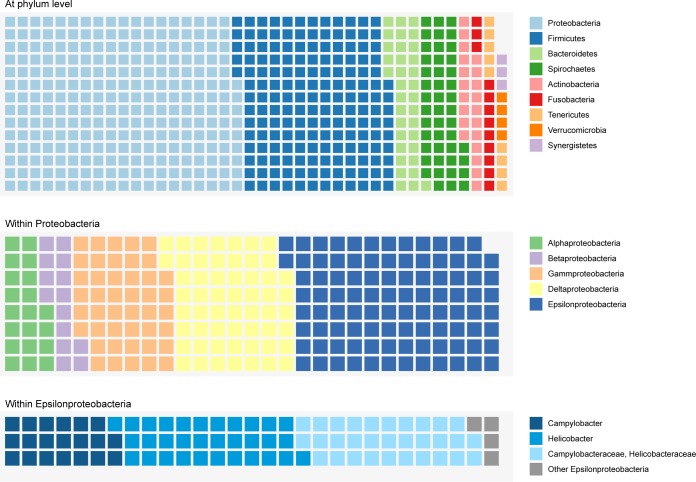
Putative sources of interphylum horizontal gene transfer in the *M. schaedleri* ASF 457 AYGZ genome. Each block represents a putative horizontally transferred gene. Blocks are colored by the predicted source of the gene and are shown at the phylum level; classes within the *Proteobacteria* and genera within the *Epsilonproteobacteria* are also shown. Nonhorizontally transferred genes are not shown.

Phylogenetic trees could be calculated for most (1,599) of the genes in the AYGZ genome. More than half of the genes have putatively been horizontally transferred between *M. schaedleri* and bacteria not belonging to the phylum *Deferribacteres*. In comparison, only 7% and 4% of genes of the genomes of the abundant gut bacteria *Bacteroides thetaiotaomicron* and *Ruminococcus bromii*, respectively, were found by the same analysis to be putative interphylum transfers. According to our analysis, many putatively horizontally transferred genes originate from *Proteobacteria* (*n* = 261) and *Firmicutes* (*n* = 168) (Fig. 3). Among *Proteobacteria*, *Epsilonproteobacteria* contributed the majority of transferred genes (*n* = 97), with the largest fraction coming from *Campylobacter* and *Helicobacter* spp. Among the *Firmicutes*, *Clostridia* contributed the largest number of genes (*n* = 108). Many of the nearest neighbors within the gene trees derived from genomes classified to the genus level as *Eubacterium* and *Clostridium*.

**FIG 4:**
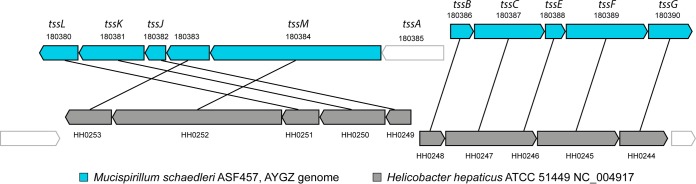
Synteny of type VI secretion system (T6SS) genes of *M. schaedleri* ASF 457 AYGZ and *Helicobacter hepaticus*. Gene names and loci are listed. TssB and TssC are components of the bacteriophage-like contractile sheath. TssE, TssJ, TssK, TssL, and TssM are components of the baseplate (97).

Most of the putative horizontally transferred genes are classified as being involved in replication, recombination, and repair (cluster of orthologous groups [COG] category L), with a large fraction coming from *Firmicutes* (Fig. S1). *Firmicutes* are also by far the largest group contributing to coenzyme transport and metabolism (COG category H) and inorganic iron transport and metabolism (COG category P), whereas *Proteobacteria* appear to be an important source for genes in most of the other COG categories. In addition, *M. schaedleri* putatively acquired several genes involved in virulence, resistance, and defense, and mobile genetic elements from other bacteria (Text S1; Fig. S1). Of note, the T6SS appears to have been horizontally transferred from *Epsilonproteobacteria*. No homologs of *M. schaedleri* genes belonging to the T6SS are present in other *Deferribacteres* genomes, and nearly all genes share a node in their phylogenetic trees with just *Campylobacter* and/or *Helicobacter* (Fig. S2); in addition, T6SS genes (minimum of 30% identity, matching at least 80% of the gene) shared between *M. schaedleri* and *Helicobacter hepaticus* had high synteny, with an almost identical gene order (Fig. 4).

10.1128/mSystems.00171-16.2 FIG S1  Potential source phyla of putatively horizontally transferred genes of *M. schaedleri* ASF 457 AYGZ grouped by cluster of orthologous groups (COG) category. Numbers of genes (A) and relative abundances of genes (B) are shown. “Other” denotes genes for which the potential source phylum is ambiguous or not listed. COG categories are chromatin structure and dynamics (B), energy production and conversion (C), cell cycle control and mitosis (D), amino acid metabolism and transport (E), nucleotide metabolism and transport (F), carbohydrate metabolism and transport (G), coenzyme metabolism (H), lipid metabolism (I), translation (J), transcription (K), replication and repair (L), cell wall/membrane/envelop biogenesis (M), cell motility (N), posttranslational modification, protein turnover, and chaperone functions (O), inorganic ion transport and metabolism (P), secondary structure (Q), general functional prediction only (R), function unknown (S), signal transduction (T), intracellular trafficking and secretion (U), and defense mechanisms (V). Download FIG S1, PDF file, 0.3 MB. Copyright © 2017 Loy et al. 2017 Loy et al. This content is distributed under the terms of the Creative Commons Attribution 4.0 International license.

10.1128/mSystems.00171-16.3 FIG S2  Phylogenetic reconstruction of genes of the type VI secretion system (T6SS) of *M. schaedleri* ASF 457 AYGZ. *M. schaedleri* is highlighted in blue. Multiple *M. schaedleri* tips indicate duplicated copies of the gene. The displayed subtrees show the phylogenetically closest group of organisms for each gene of the T6SS, and arrows indicate outgroups. Download FIG S2, PDF file, 0.4 MB. Copyright © 2017 Loy et al. 2017 Loy et al. This content is distributed under the terms of the Creative Commons Attribution 4.0 International license.

### Physiological experiments.

**FIG 5:**
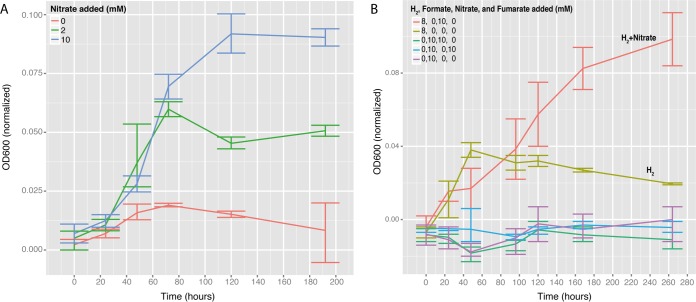
Physiological experiments of *M. schaedleri* ASF 457 MCS. Pure cultures were grown in AMM with various amounts of nitrate (A) or combinations of nitrate, formate, fumarate, and hydrogen (B). Growth was determined spectrophotometrically (at 600 nm) and was normalized by subtracting the background absorbance of the medium. Means and standard deviations of results from three replicate experiments are shown.

We tested selected features of the genome predictions in pure-culture physiological experiments with *M. schaedleri* ASF 457 MCS. Addition of nitrate significantly boosted the growth of *M. schaedleri*, and nitrate was completely reduced to ammonium, with no detectable nitrite accumulation, indicating that *M. schaedleri* is indeed capable of DNRA (Fig. 5A). To determine whether increased growth was due to nitrate reduction or to additional ammonium as a nitrogen source, *M. schaedleri* was incubated with different combinations of nitrate and/or ammonium. While ammonium alone could not elevate growth, it also did not inhibit growth in combination with nitrate (Fig. S3A). Fumarate reduction to succinate, which was also predicted by the genome, was also confirmed (Fig. S3B). Incubations with combinations of H_2_ or formate as an electron donor and nitrate and fumarate as electron acceptors were also performed. H_2_ supported growth, but no growth was detected for cultures with formate as the electron donor, even in the presence of nitrate or fumarate (Fig. 5B).

10.1128/mSystems.00171-16.4 FIG S3  Growth of *M. schaedleri* in the presence of additional ammonium (A) or fumarate (B). Growth was determined spectrophotometrically (at 600 nm) and was normalized by subtracting background absorbance from the medium. Means and standard deviations of results from three replicate experiments are shown. Download FIG S3, PDF file, 0.4 MB. Copyright © 2017 Loy et al. 2017 Loy et al. This content is distributed under the terms of the Creative Commons Attribution 4.0 International license.

### Gene expression in gnotobiotic and conventional mice.

Published metatranscriptomes from the intestinal microbiota of conventional mice were screened for reports of *Mucispirillum*. Reads from metatranscriptomes containing *Mucispirillum* spp. were then mapped to the AYGZ genome, as it has slightly more genes. Putative *M. schaedleri* transcripts were detected in 48 libraries from three studies and included cecum, colon, or pooled cecum and colon contents of healthy mice (57), dextran sulfate sodium (DSS)-treated mice (58), and nonobese diabetic mice (59). Transcripts were detected at relatively low abundance in all samples (median, 515 reads; range, 4 to 11,910 reads), which is consistent with the typically low abundance of *Mucispirillum* organisms. In total, reads mapped to 851 genes (37% of predicted genes in the genome). Among the most highly expressed were genes involved in DNRA as well as cytochrome *c* biogenesis, though not the hydrogenase 2 or the T6SS gene (Table S2).

10.1128/mSystems.00171-16.7 TABLE S2  Metatranscriptome read counts. Download TABLE S2, XLSX file, 0.4 MB. Copyright © 2017 Loy et al. 2017 Loy et al. This content is distributed under the terms of the Creative Commons Attribution 4.0 International license.

The published study with the largest number of mapped reads compared cecum and colon metatranscriptomes (59), and we attempted to determine whether there was differential gene expression in *M. schaedleri* between these two intestinal compartments. No differential gene expression was detected, though this may have been due to limited sequencing depth (median, 694 reads; range, 195 to 4,984 reads). The properties of the mucus layer are different between these two compartments (60), so to further explore whether there is differential expression of *M. schaedleri* genes in the cecum and colon, we compared levels of gene expression in gnotobiotic mice colonized with a consortium of four ASF species (ASF^4^) and *M. schaedleri*. Between 1.2 and 3% of mapped reads were mapped to *M. schaedleri*, with no statistically significant difference in relative abundances between the two compartments (median, 68,800 reads; range, 38,570 to 193,800 reads). Transcripts from 2,015 genes (87% of all genes) were detected in the RNA sequencing (RNA-seq) libraries. Among the most-expressed genes included those involved in DNRA, hydrogenase 2, the oxidative-stress response (rubrerythrin and catalase), and the T6SS. Five *M. schaedleri* genes were detected as differentially expressed (all upregulated) in the colons of the gnotobiotic animals relative to in their ceca (Table S3A). All five genes have unknown functions, though three are predicted to be exported.

10.1128/mSystems.00171-16.8 TABLE S3  Differentially expressed genes of *M. schaedleri*. (A) Differentially expressed genes of *M. schaedleri* ASF 457 MCS between the ceca and colons of ASF^4^ mice. (B) Differentially expressed genes of *M. schaedleri* ASF 457 AYGZ during acute colitis of ASF^8^ mice. The log_2_-transformed fold change (FC) is listed as well as the average log-transformed counts per million reads (logCPM). *P* values were adjusted using the false-discovery rate (FDR) method. The gene annotation is also listed. Download TABLE S3, PDF file, 0.4 MB. Copyright © 2017 Loy et al. 2017 Loy et al. This content is distributed under the terms of the Creative Commons Attribution 4.0 International license.

*Mucispirillum* has been reported to be elevated in abundance during intestinal inflammation (22, 23, 61), and we therefore evaluated its gene expression using a gnotobiotic DSS colitis mouse model harboring the eight strains of the ASF (ASF^8^). As expected, DSS treatment induced colitis, including significant weight loss, colonic shortening, and gross lesions compared to conditions for control mice (Fig. S4). RNAs from pooled cecal and colon contents were sequenced, and between 1.3 and 4.1% of mapped reads were mapped to *M. schaedleri* (median, 81,760 reads; range, 55,300 to 286,700 reads). Transcripts from 2,036 genes (89% of all genes) were detected overall. Among the most highly expressed genes included those involved in DNRA, cytochrome *c*, hydrogenase 2, and the oxidative-stress response (rubrerythrin, catalase, and superoxide dismutase). Surprisingly, only 12 genes were differentially expressed during inflammation, 11 of which were upregulated and 1 of which was downregulated (Table S3B). Genes in the putative type IVA secretion system were upregulated, as were genes encoding an uncharacterized reductase system with homology to dimethyl sulfoxide (DMSO) reductase of *Escherichia coli* and the tetrathionate reductase and trimethylamine-*N*-oxide reductase of *Salmonella enterica* serovar Typhimurium (32 to 36% identity) (AYGZv1_260003 and AYGZv1_260004, respectively).

10.1128/mSystems.00171-16.5 FIG S4  Induction of acute colitis in the dextran sodium sulfate (DSS) mouse model. Boxplots of normalized body weight (A), colon length (B), and gross score at necropsy (C) are shown. All parameters were significantly affected by DSS treatment (*P* < 0.05 for each). Download FIG S4, PDF file, 0.2 MB. Copyright © 2017 Loy et al. 2017 Loy et al. This content is distributed under the terms of the Creative Commons Attribution 4.0 International license.

### Mouse mucosal tissue gene expression.

To test whether *M. schaedleri* affects host physiology, we compared the transcriptional profiles of the cecal mucosal tissues of ASF^4^ mice with or without *M. schaedleri* ASF 457 MCS using microarray technology. Gene set enrichment analysis (GSEA) revealed that the presence of *M. schaedleri* was associated with the selective transcription of several gene sets, including those for upregulation of translation and respiratory electron transport, as well as chemokine receptors and chemokines (Table S4A). Gene sets associated with the complement and coagulation cascades, lipoprotein metabolism, and mitosis were downregulated (Table S4B). Upstream regulator analysis predicted several regulators that may be activated (such as NF-κB and PPAR-delta) or inhibited (such as epidermal growth factor [EGF]) by *M. schaedleri* (Table S5).

10.1128/mSystems.00171-16.9 TABLE S4  Differentially expressed gene sets in the ceca of mice colonized with *M. schaedleri* MCS. (A) Upregulated gene sets; (B) downregulated gene sets. All mice had an ASF^4^ microbiota as a background (6 mice per group). Size indicates the number of genes in the gene set. Differential expression is quantified by normalized enrichment scores (NES), and statistical significance is determined using the FDR-corrected *q* values. Download TABLE S4, PDF file, 0.3 MB. Copyright © 2017 Loy et al. 2017 Loy et al. This content is distributed under the terms of the Creative Commons Attribution 4.0 International license.

10.1128/mSystems.00171-16.10 TABLE S5  Predicted factors responsible for the regulation of the differentially expressed genes in mice colonized with *M. schaedleri* MCS. All mice had an ASF^4^ microbiota as a background (6 mice per group). The differential expression levels of the listed target genes were used to predict whether an upstream regulator was activated or inhibited. The differential levels of expression of the predicted upstream regulator are shown (expected [Exp.] fold change). Activation *z* score and the associated *P* values are based on a null model of no association based on a random permutation of expression levels. Download TABLE S5, PDF file, 0.3 MB. Copyright © 2017 Loy et al. 2017 Loy et al. This content is distributed under the terms of the Creative Commons Attribution 4.0 International license.

## DISCUSSION

### Metabolic strategies of *M. schaedleri.*

*M. schaedleri* has an extremely limited repertoire of carbohydrate degradation machinery, with just 3 glycoside hydrolases (family 57 α-amylases) that are likely used for processing the storage compound glycogen (see Text S1 in the supplemental material). The absence of specialized glycan-degrading enzymes was unexpected, as *M. schaedleri* inhabits a mucus layer composed of abundant complex glycoproteins, such as mucin. In comparison, gut polysaccharide degraders, such as *Bacteroides* spp., have on average 137 glycoside hydrolases (62), and *Akkermansia muciniphila*, a dedicated intestinal mucin degrader, has 35 predicted glycoside hydrolases (63). It therefore seems that *M. schaedleri* is not a primary degrader of host-derived glycans and has limited capacities to utilize dietary polysaccharides. The genome predicts that *M. schaedleri* rather uses monosaccharides, oligopeptides, amino acids, glycerol, and short-chain fatty acids (SCFAs) as the substrates for its energy metabolism (Fig. 2). It is therefore likely a consumer of breakdown products produced by hydrolytic/fermentative microorganisms, such as *Bacteroidaceae* and *Ruminococcaceae* species (62). *M. schaedleri* also has a hydrogenase (*hyb*), and addition of H_2_ in pure cultures dramatically improved its growth (Fig. 5B). As hydrogenases 3 and 4 were not found and the addition of formate did not improve growth in pure cultures (Fig. 5B), it is unlikely that *M. schaedleri* produces H_2_. It is therefore probably dependent on cross-feeding of H_2_ produced by other fermentative species, analogously to how Salmonella Typhimurium is dependent on microbiota-derived H_2_ for establishment in the gut (64). Future colocalization and coculture studies are needed to provide insights into whether *M. schaedleri* is preferably associated with certain polysaccharide-degrading species in the mucus layer, as these species may provide it with nutrients such as monosaccharides, amino acids, and H_2_.

Nitrate is an important electron acceptor in the gut, particularly during inflammation, when levels are increased due to release of nitrogen radicals from the oxidative burst (65). *M. schaedleri* can utilize nitrate as a terminal electron acceptor via dissimilatory reduction of nitrate to ammonia (DNRA) using the periplasmic enzyme NapA for conversion of nitrate to nitrite and NrfA for reduction of nitrite to ammonia (66). In addition to nitrate reduction, *M. schaedleri* has genes for a fumarate reductase that converts fumarate to succinate and encodes a C_4_-dicarboxylate transport/antiport system (*dcuAB*), which is necessary for anaerobic respiration with fumarate. In pure-culture experiments, the addition of nitrate or fumarate substantially enhanced the growth rate and yield (Fig. 5), suggesting that nitrate may partially fuel the *Mucispirillum* blooms observed during inflammation (22).

### Resistance to oxidative stress.

The intestinal mucosa is thought to be micro-oxic, and additionally, reactive oxygen and nitrogen species are increased during inflammation (67, 68). *M. schaedleri* has several systems for scavenging oxygen and reactive oxygen species, which may explain its persistence and increased relative abundance in the inflamed gut (22). Besides encoding superoxide reductase, catalase, and cytochrome *c* oxidase, the genome encodes rubrerythrin, an oxidative-stress response protein that acts as a hydrogen peroxidase reductase (69). *M. schaedleri* therefore seems to be well adapted to the micro-oxic conditions at the mucosa and in the elevated-redox environment in the gut during inflammation.

### Secretome and possible interactions with the host.

Protein secretion is used by bacterial pathogens as well as symbionts for mediating interactions with their hosts. We detected a eukaryote-like phospholipase D protein, a member of the type VI lipase effector superfamily that targets bacterial and eukaryotic membranes (52). It is possible that *M. schaedleri* uses its T6SS to antagonize other bacteria or for promoting the establishment of a mutualistic or pathogenic relationship with its host (70). The T6SS of *M. schaedleri* has probably been laterally transferred from either *Helicobacter* or *Campylobacter*, and the gene order is the same as in *H. hepaticus*, a spiral-shaped pathogen that also inhabits the murine intestinal mucus layer and plays an important role in the development of severe inflammatory bowel disease (71). Interestingly, the presence of the T6SS in *H. hepaticus* limits intestinal inflammation (72). It has yet to be shown whether the presence of *M. schaedleri* affects inflammation status or disease susceptibility. Microarray data, however, suggest that the presence of *M. schaedleri* does modify host mucosal tissue gene expression, and it appears to have proinflammatory properties (Tables S4 and S5). *M. schaedleri* also has several putative effector proteins with eukaryote-like domains, namely, ANK repeats and TPR-containing proteins, that can be used for interactions with the host and may also play a role during inflammation (73). Future studies are needed to establish whether *M. schaedleri* can act as a pathobiont, a member of the microbiota present in healthy hosts but able to alter susceptibility to inflammatory bowel disease or enteric infection (74), or is rather a commensal that benefits from the altered gut environment during inflammation.

### Putatively horizontally transferred genes.

Horizontal gene transfer (HGT) is a major source of phenotypic innovation and a way to facilitate niche adaptation. The amount of newly acquired genes in a bacterial genome is on average less than 15% (75, 76), though interphylum HGT is thought to occur more frequently in anaerobic bacteria (77). Microbiota perturbations and intestinal inflammation can, however, boost the frequency of HGT (78). More than half of the genes in the *M. schaedleri* genome were putative interphylum-transferred genes, which is much greater than the percentage of other abundant gut bacteria transferred. Many of these genes were not related to metabolic capacity but rather to features that may enhance survival and competitive growth in a selective mammalian gut environment. In particular, these genes are involved in interactions with other bacteria or the host (e.g., T6SS), resistance and defense (e.g., CRISPR, drug resistance), and mobile genetic elements. Horizontally transferred pathways like the T6SS and glycerol-3-phosphate utilization might be especially important for facilitating survival and establishment in the mammalian intestinal tract. Interestingly, several genes involved in chemotaxis, motility, and conjugation (those for T4P, T4ASS, and Tra conjugal transfer proteins) were putatively acquired via HGT. *Proteobacteria*, one of the core phyla in the mammalian gut, were the largest phylogenetic group contributing to the gene pool of *M. schaedleri*, and this was dominated by genes shared with the epsilonproteobacterial families *Helicobacteraceae* and *Campylobacteraceae*, which include inflammation-inducing enteric pathogens. Many of these genes are involved in pathogenicity and/or host interaction, which suggests that HGT contributes significantly to the putative pathobiont lifestyle of *M. schaedleri*.

### Conclusions and outlook.

Comprehensive study of *M. schaedleri* revealed that this mucus-associated bacterium is adapted to the high-redox environment of the mucus layer and is well equipped to handle oxidative bursts that occur during inflammation. In stark contrast to characterized mucus degraders, *M. schaedleri* has virtually no capacity to degrade complex polysaccharides. It therefore likely specializes in the utilization of small molecules. An exceptionally large number of genes were putatively horizontally transferred from other gut bacteria and particularly from members of the *Proteobacteria*, which are generally facultative anaerobes, inhabit the same gut microenvironment, and can tolerate high-reduction-potential conditions. This genome evolution led to the acquisition of a range of molecular mechanisms and effector proteins for interactions with the host. These genomic features, as well as the ability of *M. schaedleri* to modulate gene expression of immune-related genes, suggest that *M. schaedleri* may indeed be a pathobiont for certain diseases. Our analyses did not suggest any explanation for why *M. schaedleri* would not survive in the human gut, and it may be that its niche is already occupied by *Epsilonproteobacteria* such as *Helicobacter* spp., which are slightly better adapted to the human gut due to long-term coevolution (79).

## MATERIALS AND METHODS

### DNA sequencing and assembly.

*Mucispirillum schaedleri* ASF 457 variant MCS was provided to Bärbel Stecher by Charles River Laboratories, Inc. (Wilmington, MA, USA). Nucleic acids were extracted using a phenol-chloroform-based extraction method (80). DNAs were sequenced using the Illumina HiSeq2000 with 3-kb mate pair libraries and MinION technology (Oxford Nanopore Technologies, Oxford, United Kingdom). Illumina data were quality filtered with prinseq-lite (81), and MinION data were filtered with poretools (82) and prinseq-lite. *De novo* genome assembly was performed using SPAdes (83).

### Genome annotation.

The MCS genome and the genome of *M. schaedleri* ASF 457 variant AYGZ (84) were annotated with the MicroScope Microbial Genome Annotation and Analysis Platform (85). Metabolic pathways were reconstructed using the MicroCyc and the KEGG (86) classification schemes within MicroScope. Further details about genome analysis are provided in Text S1.

### Physiological studies.

Unless otherwise stated, *M. schaedleri* ASF 457 MCS was cultured under anaerobic conditions under an N_2_ and 8% H_2_ atmosphere at 37°C without shaking using anaerobic *Mucispirillum* medium (AMM), which is based on Trypticase soy agar and contains (per liter) 18 g brain heart broth (Merck), 15 g tryptone soy broth (Oxoid), 5 g yeast extract (Bacto yeast extract), 2.5 g K_2_HPO_4_ (Carl Roth), 1 mg hemin (Sigma), 0.5 mg vitamin K_1_ (Carl Roth), 0.4 g Na_2_CO_3_ (Carl Roth), 3% fetal calf serum (Sigma), 0.5 mg l-cysteine hydrochloride (Sigma), and 0.5 mg alpha-(d+)-glucose monohydrate (Carl Roth) (87). *M. schaedleri* ASF 457 MCS was analyzed for growth in AMM with or without the presence of the following compounds: hydrogen (8%), formate (0.5, 2.5, 10, or 50 mM), nitrate (2 or 10 mM), and fumarate (10 or 50 mM). Growth was quantified by optical density measured at 600 nm (OD_600_) (M107 high-specification visible spectrophotometer; Spectronic Camspec Ltd., Leeds, United Kingdom). OD_600_ values were normalized by subtracting the background absorbance values of abiotic-medium controls.

### Animal experiments.

In order to evaluate the gene expression of *M. schaedleri* MCS in the cecum and colon, 4- to 6-week-old C57BL/6 mice harboring a reduced altered Schaedler flora (ASF 356, ASF 361, SB2 [a reisolate of ASF 502], and ASF 519 [ASF^4^]; *n =* 3) were inoculated with *M. schaedleri* MCS and housed under gnotobiotic conditions in gnotocages. Ten days after inoculation, mice were euthanized and cecum and colon contents were collected separately and immediately frozen in liquid nitrogen for subsequent RNA-seq analysis. Animal experiments were approved by the Regierung von Oberbayern, Germany, and the local ethics committee.

To evaluate the gene expression of *M. schaedleri* during acute intestinal inflammation, age- and sex-matched 8-week-old C57BL/6 mice harboring the 8 taxa of the altered Schaedler flora (17) were maintained under gnotobiotic conditions and treated with 3% dextran sodium sulfate (DSS; molecular weights [MW], 36,000 to 50,000; MP Biomedicals, Solon, OH, USA) in the drinking water for 5 days (*n =* 8) and then given regular drinking water for another 3 days. Control animals (*n =* 8) were given drinking water without DSS for the entire study. Three days after DSS treatment, all animals were euthanized; cecal contents were collected and immediately frozen in liquid nitrogen for RNA-seq analysis. To assess disease severity, colon lengths and scores (0 to 5) were recorded at necropsy based on the presence (+1) or absence (0) of enlarged cecal tonsils, cecal atrophy, intestinal emptying, mucoid contents, and blood (modified from reference 88). All animal experiments were approved by the Institutional Animal Care and Use Committee at the University of Nebraska—Lincoln.

### RNA-seq and metatranscriptomic analysis.

Nucleic acids were extracted from collected samples, and DNase was digested twice and checked to be DNA free using PCR. rRNA was removed using the Ribo-Zero bacterial kit (Illumina, San Diego, CA) and evaluated using an RNA HighSens kit (Experion, Hercules, CA). RNA was prepared for multiplexed Illumina RNA-seq (NEBNext Ultra RNA library prep kit for Illumina with NEBNext multiplex oligonucleotides; New England Biolabs, Ipswich, MA) and sequenced on the HiSeqV4 SR100 platform (Campus Science Support Facilities GmbH, Vienna, Austria). Sequence data are available at the European Nucleotide Archive (ENA) under BioProject no. PRJEB13534. Published metatranscriptomic data sets from mice with detectable levels of *Mucispirillum* were downloaded from the National Center for Biotechnology Information Short Read Archive database and quality filtered using Trimmomatic (89). Reads were mapped to the *M. schaedleri* genome using BWA (90) and analyzed with HTSeq (91).

### Mouse microarray analysis.

C57BL/6 mice harboring a reduced altered Schaedler Flora (ASF^4^; *n =* 6) or ASF^4^ mice colonized with *M. schaedleri* MCS for 10 days (*n =* 6) were sacrificed. The cecum was washed in phosphate-buffered saline (PBS) to remove contents and stored in RNAlater (Qiagen). RNA was purified from cecal tissue samples using TRIzol (Life Technologies, Inc., Carlsbad, CA, USA) followed by RNeasy Microkit columns (Qiagen, Venlo, the Netherlands). RNA quality was assessed on the Agilent 2100 bioanalyzer (Agilent Technologies, Amsterdam, the Netherlands). RNA was labeled using an Affymetrix WT plus reagent kit and hybridized to GeneChip Mouse Gene 1.1 ST arrays (Affymetrix, Santa Clara, CA). Sample labeling, hybridization to chips, and image scanning were performed according to the manufacturer’s instructions. Microarray analysis was performed using the MADMAX pipeline (92). Quality control was performed, and all arrays met our criteria. A custom annotation that combines all individual probes for a gene (93) was used. Expression values were calculated and normalized using the robust multichip average (RMA) method (94), and significant differences were assessed using the paired intensity-based moderated T statistic (IBMT) (95). Pathway analysis was performed by gene set enrichment analysis (96); upstream regulator analysis (Ingenuity) was also performed.

### Accession number(s). 

Genome and RNA-seq data are available at the European Nucleotide Archive (ENA) under BioProject no. PRJEB13534, and microarray data are available at the NCBI GEO repository under accession no. GSE83625.
