# Supplementary material for: Lifestyle and Horizontal Gene Transfer-Mediated Evolution of Mucispirillum schaedleri, a Core Member of the Murine Gut Microbiota
Source: mSystems. 2017 Jan 31;2(1):e00171-16. doi: 10.1128/mSystems.00171-16 (PMC5285517; doi:10.1128/mSystems.00171-16)
Supplement: TABLE S1 [file sys001172082st6.pdf]

Table S1. General features of the genomes of *Mucispirillum schaedleri* ASF 457 sub-strains AYGZ and MCS.

	AYGZ	MCS
Total size (Mb)	2.3	2.3
GC content	31.15%	31.16%
Number of contigs	39	36
Repeated Regions	10.54%	11.08%
Average CDS length (bp)	923.71	931.26
Average intergenic length (bp)	147.5	146.77
Protein coding density	88.21%	87.94%
# of genomic objects without artifacts	2,285	2,271
Total # of CDS	2,227	2,218
Protein coding genes	2,223	2,214
5S / 16S / 23S rRNA	2 / 3 / 2	2 / 2 / 2
tRNA	39 (complete set)	38 (complete set)
Housekeeping genes detected (estimate of completeness)	31 (of 31)	31 (of 31)
