# Supplementary material for: Lifestyle and Horizontal Gene Transfer-Mediated Evolution of Mucispirillum schaedleri, a Core Member of the Murine Gut Microbiota
Source: mSystems. 2017 Jan 31;2(1):e00171-16. doi: 10.1128/mSystems.00171-16 (PMC5285517; doi:10.1128/mSystems.00171-16)
Supplement: TEXT S1 [file sys001172082s1.pdf]

Supplementary Methods

Genome Completeness Check

The completeness check for both genomes was done using tRNAscan-SE (version 1.23) (Lowe & Eddy, 1997), RNAmmer (version 1.2) (Lagesen *et al.*, 2007), AMPHORA2 (Wu & Scott, 2012), and CheckM (lineage workflow) (Parks *et al.*, 2015).

Type VI secretion system (T6SS)

The type VI secretion system (T6SS) consists of a gene cluster containing 13 core genes (Boyer *et al.*, 2009). These core components have distinct Clusters of Orthologous Groups (COG) IDs, of which most are unique for T6SS function. For characterization of the T6SS these COG IDs were used to search for in MicroScope. Gene names were taken from UniProt. T6SS cluster genes with missing COG ID annotation in MicroScope were identified by searching the NCBI NR dataset using Protein BLAST.

Clustered Regularly Interspaced Short Palindromic Repeats (CRISPR) system and mobile genetic elements identification

CRISPR repeats were identified using the CRISPR recognition tool (CRT) (Bland *et al.*, 2007) as well as Piler-CR (Edgar, 2007). Potential viruses corresponding to spacers within the CRISPR loci were searched for using CRISPRTarget (Biswas *et al.*, 2013) by predicting and analyzing crRNA targets. CRISPRTarget was run with CRT results with default parameters (database: Genbank-Phage, RefSeq-Plasmid; gap open: -10; gap extend: -2; nucleotide match: 1; nucleotide mismatch: -1; e-value: 1; word size: 7). Identification of the CRISPR array position in the genome and of the accompanying c*sn* and *cas* genes was done manually in MicroScope. Insertion sequences (IS) were identified using ISfinder (Siguier *et al.*, 2006). Protein sequences were blasted against the IS database, which provides a list of insertion sequences from bacteria and

archaea, with BLASTP (version 2.2.31+) (Camacho *et al.*, 2009) with a custom log e-value of -3. IS families of genes producing significant alignments were extracted from the BLAST results. PHAST (Zhou *et al.*, 2011) was used for the similarity based analysis of putative prophages and phage-like proteins, whereby genomic regions that are enriched in protein coding genes with known phage homologs are detected.

Proteins with eukaryotic-like domains

For identification of proteins with Ankyrin repeat (ANK) domains, the PROSITE (Sigrist *et al.*, 2002; 2012) motif for ANK_REP_REGION was used to scan it against the genomes using ScanProsite web service (option 3) (de Castro *et al.*, 2006). Effective 4.0 (http://www.effectors.org) (Jehl *et al.*, 2011) was used to predict other secreted bacterial proteins. To obtain domain names, amino acid sequences of predicted loci were extracted and scanned against prosite.dat (release: 10.112 of Mar 04, 2015) using the Perl script ps_scan.pl (version: 1.79). Both files were obtained from the PROSITE website ftp://ftp.expasy.org/databases/prosite/. The results were manually inspected, processed and sorted.

Identification of non-identical genes

Non-identical genes between the two strains were identified by subtracting almost identical genes (homology constraints were minLrap ≥ 0.9, maxLrap ≥ 0.9, identity ≥ 100%) from all genes (homology constraints were minLrap ≥ 0.9, maxLrap ≥ 0.9, identity ≥ 30%) using MicroScope's Gene Phyloprofile interface, whereby minLrap is defined as the quotient of the length of the match and the length of the shorter protein, whereas maxLrap is defined as the quotient of the length of the match and the length of the longer protein.

Putative horizontally-transferred genes

For horizontal gene transfer (HGT) analysis phylogenetic trees for each gene were calculated using the software PhyloGenie (Frickey & Lupas, 2004). Trees were constructed using RAxML version 8.2.4 with the GAMMA model of rate heterogeneity and rapid bootstrapping (100 bootstraps) (Stamatakis, 2014). To identify putative HGT events, we collected trees containing a node connecting *Mucispirillum* exclusively with a specified phylogenetic group but with no other groups. The trees that met this criterion were selected using PHAT (part of the PhyloGenie software package). Phylogenetically-closest species in each tree were identified using R (version: 3.2.1) (Team, 2013) and the R packages phytools (version: 0.4-56) (Revell, 2012) and ape (Paradis *et al.*, 2004) by extracting species with the minimum phylogenetic distance within the node containing *M. schaedleri*.

Supplementary Results

Genome reconstruction and comparison

Recently, the genome of *M. schaedleri* ASF 457 (genome AYGZ) from a culture maintained in an American collection was announced (Wannemuehler *et al.*, 2014). We had meanwhile sequenced the genome of *M. schaedleri* ASF 457 (genome MCS) using a culture maintained in a strain collection in Germany. While we believe that the two cultures are originally derived from the same stock from the Charles River laboratories, it is unclear how long the two cultures have been separated and how many bacterial generations may have occurred subsequently. Neither genome is closed, but estimates based on detection of tRNAs and conserved housekeeping genes indicate that the genomes are largely complete (Table S1). Both genomes have a GC content of 31% and a coding density of 88%. The number of detected coding sequences (CDS) without artifacts is 2,227 for the AYGZ and 2,218 for the MCS genome. The content of genomic objects in the two genomes is highly similar, with shared CDSs accounting for 92% of all CDSs in both genomes (shared CDSs are defined as having ≥ 80% amino acid similarity and ≥ 80% sequence length). As the genomes are not closed it is not possible to determine whether the

differences in CDSs is due to absence from a genome or due to technical artifacts (incomplete sequencing and/or assembly).

General genomic features

Central metabolism

A complete Embden-Meyerhof-Parnas (EMP) pathway was detected in the *M. schaedleri* genome. For the first step of this pathway, the phosphorylation of glucose can be performed by either a glucokinase (E.C. 2.7.1.2) or a phosphotransferase system (PTS, E.C. 2.7.1.69). The oxidative phase of the pentose phosphate pathway was not detected, but the non-oxidative phase is present (although the transaldolase, E.C. 2.2.1.2, could not be identified in either genome), which should allow for synthesis of five carbon sugars from glycolysis. The *M. schaedleri* genome does not encode proteins for an Entner-Doudoroff pathway. Pyruvate:flavodoxin oxidoreductase (E.C. 1.2.7.-), but not pyruvate:formate lyase or pyruvate dehydrogenase, is present, which is likely involved in converting pyruvate to acetyl-CoA. A complete tricarboxylic acid (TCA) cycle could be identified, and features a *Helicobacter*-type succinyl-CoA:acetoacetate CoA transferase (SCOT, E.C. 2.8.3.8). A glyoxylate pathway (i.e. the genes for isocitrate lyase and malate synthase) was not detected. Oxaloacetate can be used to replenish the EMP pathway via the phosphoenolpyruvate (PEP) carboxykinase (E.C. 4.1.1.49). The EMP and TCA pathways can also be replenished from acetate or lactate utilization. Fructose can also be utilized via a fructokinase (E.C. 2.7.1.4).

Amino acid biosynthesis

M. schaedleri has complete biosynthetic pathways for the amino acids alanine (from cysteine), β-alanine (from aspartate), arginine (acetyl cycle), cysteine, D-glutamate, D-glutamine, glycine, homocysteine, isoleucine, leucine, phenylalanine, proline, tyrosine, and valine. Biosynthesis of aspartate from glutamate via an aspartate aminotransferase was not detected, but aspartate may be produced from fumarate via an aspartate ammonia-lyase (E.C. 4.3.1.1). Almost complete amino acid biosynthesis pathways include histidine, lysine, and ornithine. Other

incomplete or not detected pathways include asparagine, homoserine, methionine, selenocysteine, serine, threonine, and tryptophan.

Putative electron donors and carbon sources

The genome encodes 15 proteases, of which 4 (AYGZ) respectively 5 (MCS) are predicted to be secreted, and 3 aminopeptidases. Catabolic pathways for glutamine, asparagine, and cysteine are present. The genome encodes multiple ABC transporters for amino acids in general, and in particular for leucine/isoleucine/valine, methionine and toluene. It has transporters for peptides (ABC-type), oligopeptides (appBCD), and a permease for oligopeptides is also present. Also an ABC-type transporter for polyamines could be detected. The genome features an extremely reduced repertoire of polysaccharide degradation machinery with just 3 glycoside hydrolases belonging to family 57 (α-amylases).

M. schaedleri has genes for degradation of glycerophosphodiester and glycerol utilization, including a periplasmic glycerophosphodiester phosphodiesterase (E.C. 3.1.4.46), sn-glycerol-3-phosphate transporter, glycerol kinase, and both aerobic and anaerobic versions of the sn-glycerol-3-phosphate dehydrogenase (E.C. 1.1.1.94, respectively E.C. 1.1.5.3). The anaerobic flavin-dependent sn-glycerol-3-phosphate dehydrogenase is localized in the cytoplasmic membrane and couples oxidation of sn-glycerol-3-phosphate to glycerone phosphate with the reduction of quinone to quinol. Glycerol dehydrogenases are important for phospholipid biosynthesis as well as usage of glycerophosphodiesters as electron donor. The β-oxidation pathway for fatty acid degradation is incomplete. Only 1 out of 7 reactions are present (just a Long-chain fatty acid-CoA ligase E.C. 6.2.1.3 could be detected), which indicates that the pathway is likely absent. Dicarboxylate and C4-dicarboxylate as well as short-chain fatty acid transporters are encoded in the genome.

Cofactors and vitamins

M. schaedleri can produce coenzyme A (CoA), which plays a role in the oxidation and biosynthesis of fatty acids and in the TCA cycle. Biotin, a water-soluble B-vitamin that, as a

coenzyme, is involved in gluconeogenesis and in the synthesis of isoleucine, valine and fatty acids, can also be produced. It can be synthesized from 7-keto-8-aminopelargonate, from riboflavin and flavin adenine dinucleotide (FAD), an essential flavin cofactor that is involved in a variety of redox reactions, and from thiamin diphosphate, which plays an essential role in energy metabolism as a cofactor of a variety of enzymes like pyruvate dehydrogenase or transketolase. The pathway for *de novo* biosynthesis of coenzyme B12 (cobalamin coenzyme) is incomplete, but *M. schaedleri* can synthesize coenzyme B12 from cobalamin. Although no transporter for cobalamin was detected, it can probably be synthesized *de novo* from cobinamide via an uncharacterized route.

Other transporters

SecD-SecG and SecY translocases from the Sec translocase-mediated pathway, and TatA and TatC translocases from the twin-arginine translocation (Tat) system for protein translocation across and insertion into membranes were detected. *M. schaedleri* has transporters for molybdate (ABC-type), peptide/nickel (ABC-type), nickel (ABC-type), iron (ABC-type), magnesium, cobalt (ABC-type), cadmium and zinc. A sodium:proton antiporter was detected and the genome putatively also encodes a biotin transporter, a lipopolysaccharide transporter and a sulfate transporter. A drug resistance MFS transport protein (drug:H+ antiporter-2 family), a putative multidrug-efflux transporter MexB and the multidrug efflux system protein SugE for exporting antibiotics and other cytotoxic substances are also present.

Storage compounds

M. schaedleri appears to be able to produce glycogen as a storage compound, as a glycogen synthase (E.C. 2.4.1.21) and a glycogen phosphorylase (E.C. 2.4.1.1) were detected in the genome. Adjacent to the glycogen synthase there are three glycosyl hydrolases (family 57) that may be involved in glycogen processing. A polyphosphate kinase (Ppk) was detected which enables the organism to synthesize polyphosphate (poly P) from inorganic phosphorus or from the terminal phosphate of ATP. Despite the use of poly P as a potential energy source, it might

also play a role in both stress response and pathogenicity. A poly P-AMP-phosphotransferase (PAP), which phosphorylates AMP to ADP using poly P as a substrate, could not be detected.

Motility

The genome encodes more than 80 proteins classified in the COG group Cell Motility. Besides genes needed for biosynthesis of its flagellum, several chemotaxis-related proteins are present in the genome. The histidine kinase CheA, two coupling chemotaxis proteins CheW – involved in the transmission of signals from the transmembrane chemotaxis receptor proteins to the flagellar motor – and several putative methyl-accepting chemotaxis proteins (MCP) could be detected. The PseB (E.C. 4.2.1.115) protein and the pseudaminic acid synthase PseI (E.C. 2.5.1.97), catalyzing the first respectively the last step in the biosynthesis of pseudaminic acid, were detected.

Virus defense (CRISPR)

M. schaedleri has a CRISPR/Cas-System with a length of 694 nucleotides, which is identical between the two genomes. The *cas1*, *cas2* and *cas9* genes were detected, indicating that it is a type II CRISPR/Cas-System (Makarova *et al.*, 2011). There are 10 spacers, all of them identical between both genomes, and a repeat consensus sequence with a length of 36 nucleotides. We searched for targets of the crRNA spacers, but of 10 spacers only one had a single match, which was *Bacillus thuringiensis* MC28 plasmid pMC189 (NC_018687).

Mobile genetic elements

One intact prophage, including putative head and tail proteins, was detected in the AYGZ genome. All of the predicted phage-like proteins are also present within a region in the MCS genome, but have not been predicted as intact prophage. The prophage region has a size of 134.6 kb and 32% of CDS (43 total) in this region encode phage-like proteins. Consistent with known quality of integration sites, multiple tRNAs and transposases were detected in this region, although an integrase could not be identified. The putative head and tail proteins are located in a region in close vicinity to the CRISPR/Cas-system region. No plasmid was identified.

Putative horizontally transferred genes (HGT)

M. schaedleri putatively acquired several genes involved in virulence, resistance and defense, and mobile genetic elements from other bacteria. The gene of the HlyD family secretion protein, which is involved in the transport of hemolysin A shares a node with *Helicobacter*. Parts of the CRISPR/Cas-system were putatively acquired from *Bacilli* and *Epsilonproteobacteria*, with the CRISPR-associated Csn1 (Cas9) family protein coming from *Staphylococcus* (*Bacilli*) and the CRISPR-associated endonuclease Cas1 protein coming from either *Campylobacter* or *Helicobacter* (*Epsilonproteobacteria*), which also appear to be the origin of the *vapD* gene. The Tra conjugal transfer proteins and the VirB complex from a putative type 4 secretions system are related to genes from *Proteobacteria*. Genes involved in resistance appear to have its origin in a wider range of phylogenetic groups with a drug resistance MFS transporter (drug:H+ antiporter-2 family) coming from *Bifidobacterium*, a drug/metabolite transporter (DMT family) coming from *Staphylococcus*, a putative multidrug resistance protein MexB coming from *Desulfovibrionaceae*, and a putative β-lactamase coming from *Campylobacter*. The putative methyl-accepting chemotaxis protein is related to genes from *Campylobacter*. *Clostridia* appear to be the source of several proteins involved in transport, cofactor biosynthesis, respiration and oxygen stress response. Transport proteins for cobalt are related to those from *Clostridium* as well as the nitroreductase and ruberythrin. A cobalamin synthase putatively comes from *Eubacterium*, the hydrogenase 2 from *Geobacter* and the catalase from *Sphaerochaeta*. Most of the putative HGT genes are classified in the COG classification scheme as replication, recombination and repair (COG category L) with a large fraction coming from *Firmicutes*. *Firmicutes* are also by far the largest group putatively contributing to coenzyme transport and metabolism (COG category H) and inorganic iron transport and metabolism (COG category P), whereas *Proteobacteria* appear to be an important source for laterally transferred genes in most of the other COG categories.

References

Biswas A, Gagnon JN, Brouns SJJ, Fineran PC, Brown CM. (2013). CRISPRTarget. *RNA Biology* **10**:817–827.

Bland C, Ramsey TL, Sabree F, Lowe M, Brown K, Kyrpides NC, Hugenholtz P. (2007). CRISPR Recognition Tool (CRT): a tool for automatic detection of clustered regularly interspaced palindromic repeats. *BMC Bioinformatics 2007 8:1* **8**:1.

Boyer F, Fichant G, Berthod J, Vandenbrouck Y, Attree I. (2009). Dissecting the bacterial type VI secretion system by a genome wide in silico analysis: what can be learned from available microbial genomic resources? *BMC Genomics* **10**:1.

Camacho C, Coulouris G, Avagyan V, Ma N, Papadopoulos J, Bealer K, Madden TL. (2009). BLAST+: architecture and applications. *BMC Bioinformatics 2007 8:1* **10**:1.

de Castro E, Sigrist CJA, Gattiker A, Bulliard V, Langendijk-Genevaux PS, Gasteiger E, Bairoch A, Hulo N. (2006). ScanProsite: detection of PROSITE signature matches and ProRule-associated functional and structural residues in proteins. *Nucleic Acids Res* **34**:W362–5.

Edgar RC. (2007). PILER-CR: Fast and accurate identification of CRISPR repeats. *BMC Bioinformatics 2007 8:1* **8**:1.

Frickey T, Lupas AN. (2004). PhyloGenie: automated phylome generation and analysis. *Nucleic Acids Res* **32**:5231–5238.

Jehl M-A, Arnold R, Rattei T. (2011). Effective--a database of predicted secreted bacterial proteins. *Nucleic Acids Res* **39**:D591–5.

Lagesen K, Hallin P, Rødland EA, Stærfeldt H-H, Rognes T, Ussery DW. (2007). RNAmmer: consistent and rapid annotation of ribosomal RNA genes. *Nucleic Acids Res* **35**:3100–3108.

Lowe TM, Eddy SR. (1997). tRNAscan-SE: a program for improved detection of transfer RNA genes in genomic sequence. *Nucl Acids Res* **25**:955–964.

Makarova KS, Haft DH, Barrangou R, Brouns SJJ, Charpentier E, Horvath P, Moineau S, Mojica FJM, Wolf YI, Yakunin AF, van der Oost J, Koonin EV. (2011). Evolution and classification of the CRISPR–Cas systems. *Nature Reviews Microbiology* **9**:467–477.

Paradis E, Claude J, Strimmer K. (2004). APE: Analyses of Phylogenetics and Evolution in R language. *Bioinformatics* **20**:289–290.

Parks DH, Imelfort M, Skennerton CT, Hugenholtz P, Tyson GW. (2015). CheckM: assessing the quality of microbial genomes recovered from isolates, single cells, and metagenomes. *Genome Res* **25**:1043–1055.

Revell LJ. (2012). phytools: an R package for phylogenetic comparative biology (and other

things). *Methods Ecol Evol* **3**:217–223.

Sigrist CJA, Cerutti L, Hulo N, Gattiker A, Falquet L, Pagni M, Bairoch A, Bucher P. (2002). PROSITE: A documented database using patterns and profiles as motif descriptors. *Brief Bioinformatics* **3**:265–274.

Sigrist CJA, de Castro E, Cerutti L, Cuche BA, Hulo N, Bridge A, Bougueleret L, Xenarios I. (2012). New and continuing developments at PROSITE. *Nucl Acids Res* **41**:gks1067–D347.

Siguier P, Pérochon J, Lestrade L, Mahillon J, Chandler M. (2006). ISfinder: the reference centre for bacterial insertion sequences. *Nucleic Acids Res* **34**:D32–6.

Stamatakis A. (2014). RAxML version 8: a tool for phylogenetic analysis and post-analysis of large phylogenies. *Bioinformatics* **30**:1312–1313.

Team RC. (2013). A language and environment for statistical computing. R Foundation for Statistical Computing, Vienna, Austria.

Wannemuehler MJ, Overstreet A-M, Ward DV, Phillips GJ. (2014). Draft genome sequences of the altered schaedler flora, a defined bacterial community from gnotobiotic mice. *Genome Announc* **2**:e00287–14–e00287–14.

Wu M, Scott AJ. (2012). Phylogenomic analysis of bacterial and archaeal sequences with AMPHORA2. *Bioinformatics* **28**:1033–1034.

Zhou Y, Liang Y, Lynch KH, Dennis JJ, Wishart DS. (2011). PHAST: A Fast Phage Search Tool. *Nucl Acids Res* **39**:gkr485–W352.
