# Supplementary material for: Lifestyle and Horizontal Gene Transfer-Mediated Evolution of Mucispirillum schaedleri, a Core Member of the Murine Gut Microbiota
Source: mSystems. 2017 Jan 31;2(1):e00171-16. doi: 10.1128/mSystems.00171-16 (PMC5285517; doi:10.1128/mSystems.00171-16)
Supplement: FIG S1 [file sys001172082sf2.pdf]

A

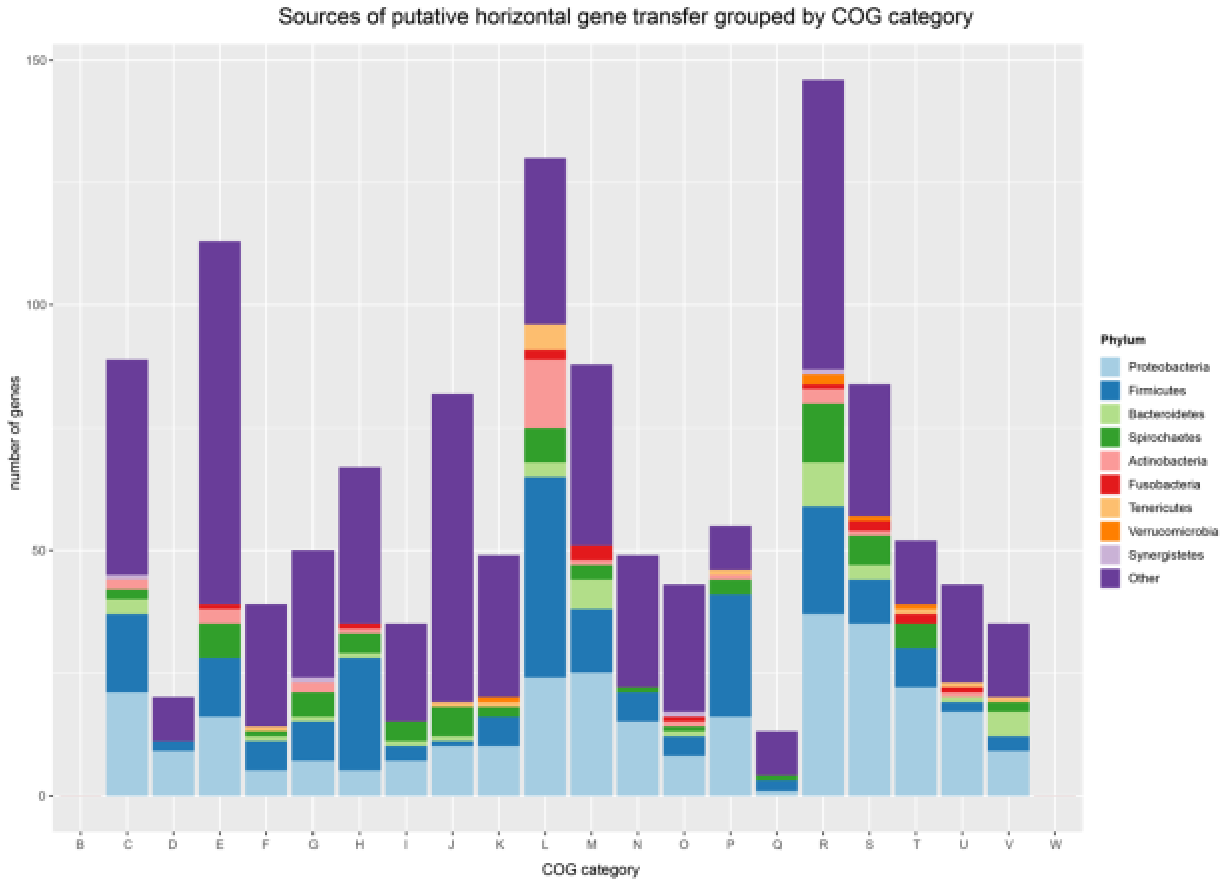

Sources of putative horizontal gene transfer grouped by COG category
number of genes
0
50
100
150
B
C
D
E
F
G
H
I
J
K
L
M
N
O
P
Q
R
S
T
U
V
W
COG category
Phylum
Proteobacteria
Firmicutes
Bacteroidetes
Spirochaetes
Actinobacteria
Fusobacteria
Tenericutes
Verrucomicrobia
Synergistetes
Other

B

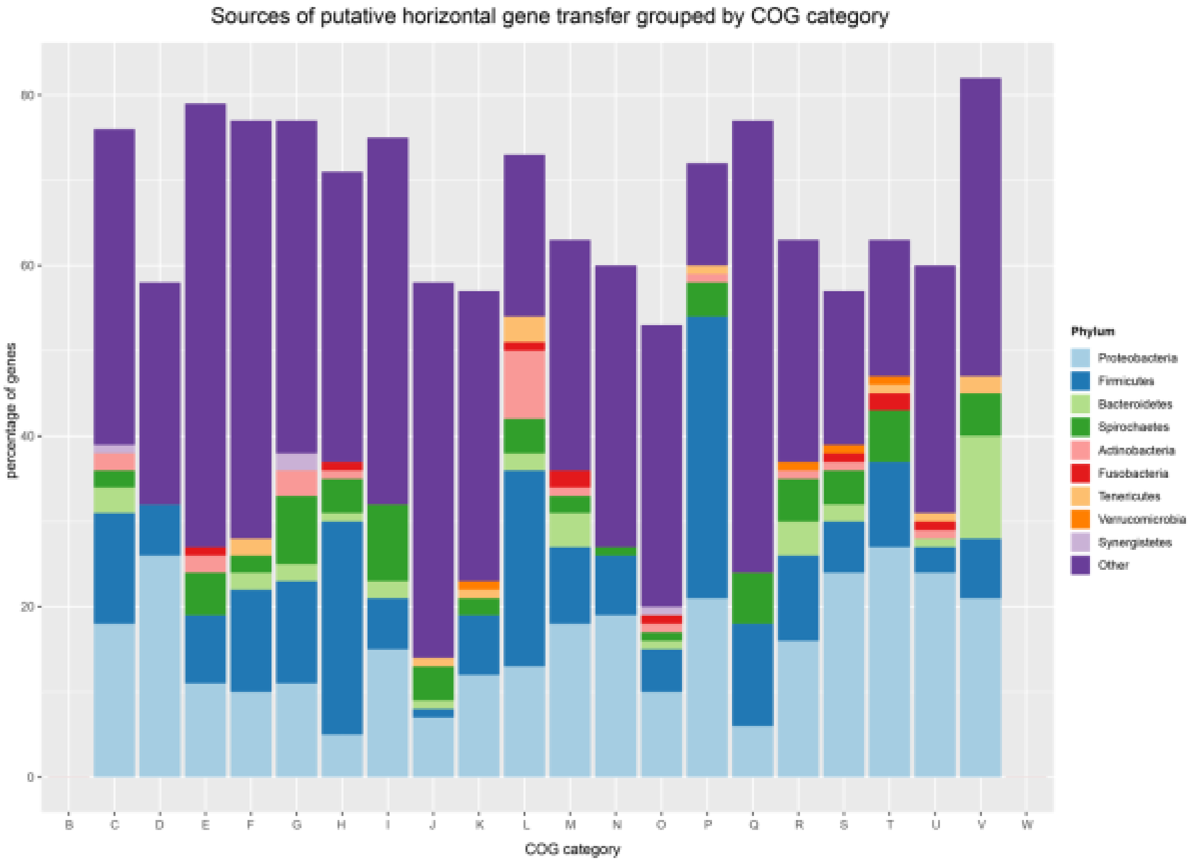

Figure S1. Potential source phylum of putatively horizontally-transferred genes of *M. schaedleri* ASF 457 AYGZ grouped by Clusters of Orthologous Groups (COG) category. (A) Number of genes and (B) relative abundance of genes are shown. “Other” denotes genes for which the potential source phylum is ambiguous or not listed. COG categories are: B: Chromatin Structure and dynamics, C: Energy production and conversion, D: Cell cycle control and mitosis, E: Amino Acid metabolism and transport, F: Nucleotide metabolism and transport, G: Carbohydrate metabolism and transport, H: Coenzyme metabolism, I: Lipid metabolism, J: Translation, K: Transcription, L: Replication and repair, M: Cell wall/membrane/envelop biogenesis, N: Cell motility, O: Post-translational modification, protein turnover, chaperone functions, P: Inorganic ion transport and metabolism, Q: Secondary Structure, R: General Functional Prediction only, S: Function Unknown T: Signal Transduction, U: Intracellular trafficking and secretion, and V: Defense mechanisms.
