# Supplementary material for: Lifestyle and Horizontal Gene Transfer-Mediated Evolution of Mucispirillum schaedleri, a Core Member of the Murine Gut Microbiota
Source: mSystems. 2017 Jan 31;2(1):e00171-16. doi: 10.1128/mSystems.00171-16 (PMC5285517; doi:10.1128/mSystems.00171-16)
Supplement: FIG S2 [file sys001172082sf3.pdf]

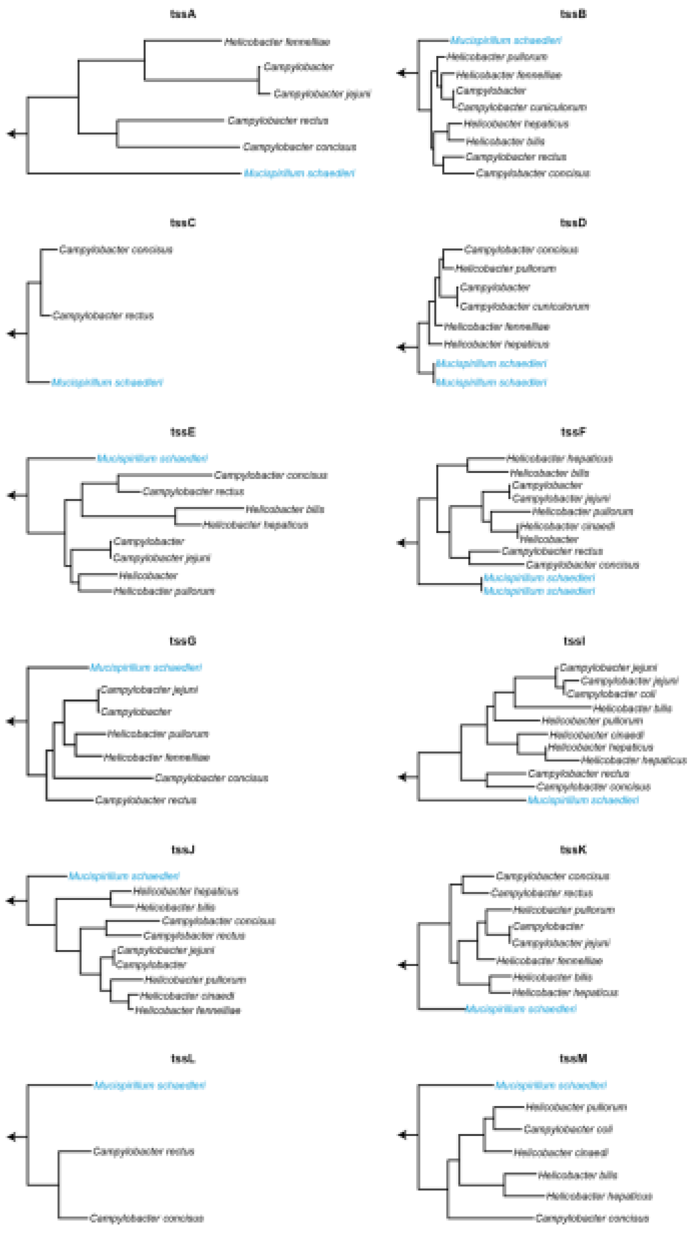

Figure S2. Phylogenetic reconstruction of genes of the Type VI Secretion System (T6SS) of *M. schaedleri* ASF 457 AYGZ. *M. schaedleri* is highlighted in blue. Multiple *M. schaedleri* tips indicate duplicated copies of the gene. The displayed sub-trees show the phylogenetically-closest group of organisms for each gene of the T6SS and arrows indicate outgroups.
