# Supplementary material for: Lifestyle and Horizontal Gene Transfer-Mediated Evolution of Mucispirillum schaedleri, a Core Member of the Murine Gut Microbiota
Source: mSystems. 2017 Jan 31;2(1):e00171-16. doi: 10.1128/mSystems.00171-16 (PMC5285517; doi:10.1128/mSystems.00171-16)
Supplement: FIG S3 [file sys001172082sf4.pdf]

A

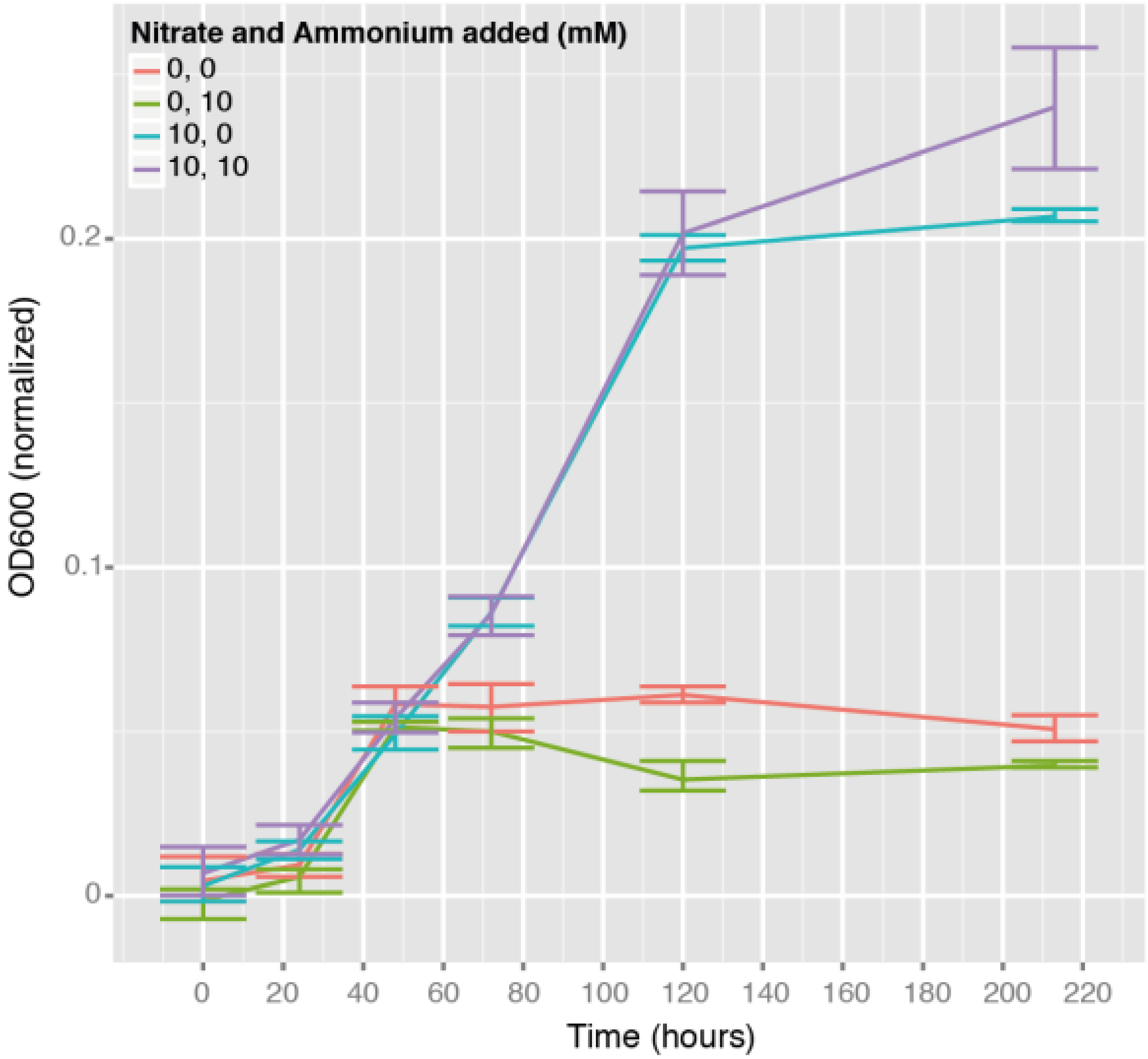

Nitrate and Ammonium added (mM)
0, 0
0, 10
10, 0
10, 10
0.2
0.1
0
OD600 (normalized)
0
20
40
60
80
100
120
140
160
180
200
220
Time (hours)

B

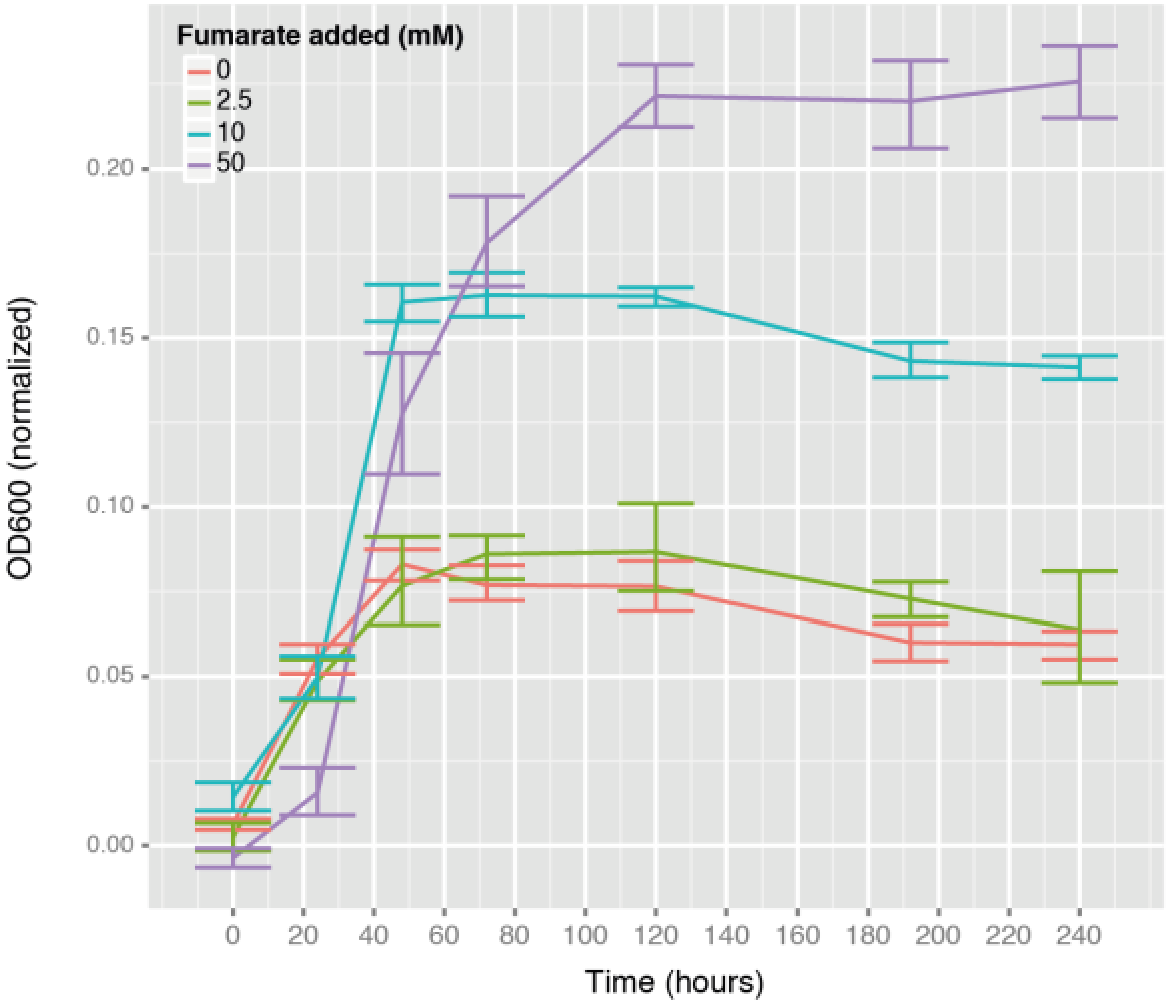

Figure S3. Growth of *M. schaedleri* in the presence of (A) additional ammonium or (B) fumarate. Growth was determined spectrophotometrically (at 600 nm) and was normalized by subtracting background absorbance from the medium. Mean and standard deviation of three replicate experiments is shown.
