# Supplementary material for: Lifestyle and Horizontal Gene Transfer-Mediated Evolution of Mucispirillum schaedleri, a Core Member of the Murine Gut Microbiota
Source: mSystems. 2017 Jan 31;2(1):e00171-16. doi: 10.1128/mSystems.00171-16 (PMC5285517; doi:10.1128/mSystems.00171-16)
Supplement: TABLE S3 [file sys001172082st8.pdf]

Table S3. Differentially-expressed genes of *M. schaedleri*. (A) Differentially-expressed genes of *M. schaedleri* ASF 457 MCS between cecum and colon of ASF4 mice. (B) Differentially-expressed genes of *M. schaedleri* ASF 457 AYGZ during acute colitis ASF8 mice. The log2-transformed fold change (FC) is listed as well as the average log-transformed counts per million reads (logCPM). P-values were adjusted using the fdr method. The gene annotation is also listed.

A

Gene	FC (colon vs. cecum)	logCPM	P-value	Annotation
MCSv2_30030	3.027	9.217	0.002	conserved exported protein of unknown function
MCSv2_30031	2.646	8.932	0.004	conserved exported protein of unknown function
MCSv2_30139	3.453	6.478	0.019	conserved protein of unknown function
MCSv2_30174	1.898	7.678	0.053	conserved exported protein of unknown function
MCSv2_30192	1.964	7.336	0.053	conserved protein of unknown function

B

Gene	FC (control vs. DSS)	logCPM	P-value	Annotation
AYGZv1_100007	3.036	5.803	0.054	protein of unknown function
AYGZv1_150075	-6.703	6.239	0.054	Conjugal transfer protein TrbB
AYGZv1_150076	-8.309	7.567	0.002	membrane protein of unknown function
AYGZv1_150077	-7.619	6.969	0.009	Conjugal transfer protein TrbD
AYGZv1_150078	-9.158	8.320	0.001	Type IV secretion/conjugal transfer ATPase, VirB4 family
AYGZv1_150080	-6.921	6.401	0.033	Mating pair formation protein TrbG
AYGZv1_150082	-7.130	6.638	0.033	protein of unknown function
AYGZv1_150083	-9.036	8.187	0.001	exported protein of unknown function
AYGZv1_150086	-4.161	8.686	0.011	protein of unknown function
AYGZv1_260003	-4.350	7.301	0.001	4Fe-4S ferredoxin iron-sulfur binding domain protein
AYGZv1_260004	-3.069	8.148	0.009	protein of unknown function
AYGZv1_tRNA31	-2.981	8.173	0.033	Methionine tRNA
