# Supplementary material for: Lifestyle and Horizontal Gene Transfer-Mediated Evolution of Mucispirillum schaedleri, a Core Member of the Murine Gut Microbiota
Source: mSystems. 2017 Jan 31;2(1):e00171-16. doi: 10.1128/mSystems.00171-16 (PMC5285517; doi:10.1128/mSystems.00171-16)
Supplement: FIG S4 [file sys001172082sf5.pdf]

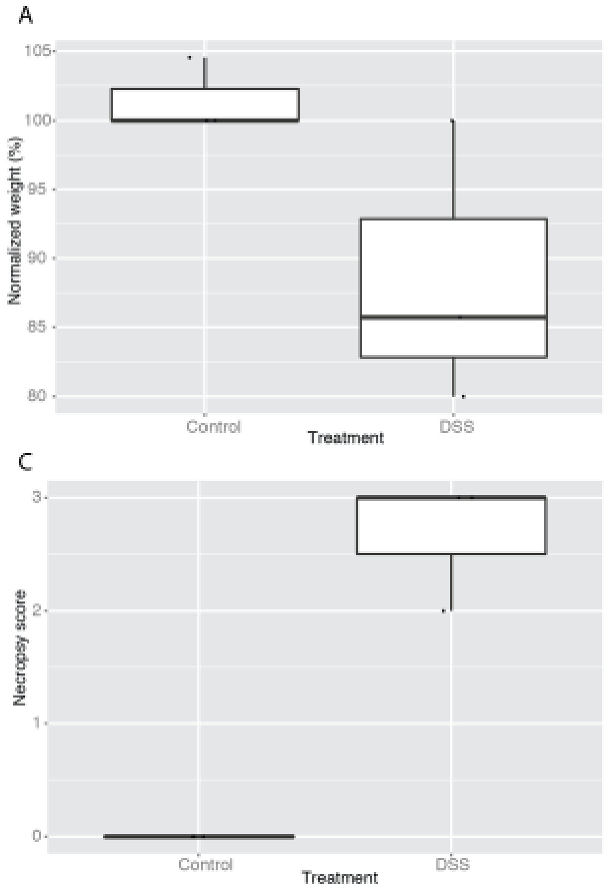

Figure S4. Induction of acute colitis in the dextran sodium sulfate (DSS) mouse model. Boxplots of (A) normalized body weight, (B) colon length, and (C) gross score at necropsy are shown. All parameters were significantly affected by DSS treatment ($p<0.05$ for each).
