# Supplementary material for: Lifestyle and Horizontal Gene Transfer-Mediated Evolution of Mucispirillum schaedleri, a Core Member of the Murine Gut Microbiota
Source: mSystems. 2017 Jan 31;2(1):e00171-16. doi: 10.1128/mSystems.00171-16 (PMC5285517; doi:10.1128/mSystems.00171-16)
Supplement: TABLE S4 [file sys001172082st9.pdf]

Table S4. Differentially-expressed gene sets in the cecum of mice colonized with *M. schaedleri* MCS. (A) Upregulated gene sets and (B) downregulated gene sets. All mice had an ASF[4] microbiota as a background (n=6 per group). Size indicates the number of genes in the gene set. Differential expression is quantified by normalized enrichment scores (NES) and statistical significance is determined using the fdr-corrected q-values.

A

Gene set	Size	NES	FDR q-val
EUKARYOTIC.TRANSLATION.ELONGATION	46	2.599	0.000
PEPTIDE.CHAIN.ELONGATION	42	2.591	0.000
EUKARYOTIC.TRANSLATION.TERMINATION	42	2.489	0.000
FORMATION.OF.A.POOL.OF.FREE.40S.SUBUNITS	52	2.468	0.000
L13A.MEDIATED.TRANSLATIONAL.SILENCING.OF.CERULOPLASMIN.EXPRESSION	60	2.410	0.000
NONSENSE.MEDIATED.DECAY.NMD.INDEPENDENT.OF.THE.EXON.JUNCTION.COMPLEX.EJC.	47	2.386	0.000
KEGG_RIBOSOME	88	2.384	0.000
X3.UTR.MEDIATED.TRANSLATIONAL.REGULATION	60	2.376	0.000
VIRAL.MRNA.TRANSLATION	45	2.351	0.000
GTP.HYDROLYSIS.AND.JOINING.OF.THE.60S.RIBOSOMAL.SUBUNIT	61	2.318	0.000
SRP.DEPENDENT.COTRANSLATIONAL.PROTEIN.TARGETING.TO.MEMBRANE	62	2.219	0.000
EUKARYOTIC.TRANSLATION.INITIATION	68	2.211	0.000
CAP.DEPENDENT.TRANSLATION.INITIATION	68	2.202	0.000
TRANSLATION	98	2.110	0.001
RESPIRATORY.ELECTRON.TRANSPORT	68	2.104	0.001
TRANSLATION.INITIATION.COMPLEX.FORMATION	37	2.100	0.001
FORMATION.OF.THE.TERNARY.COMPLEX.AND.SUBSEQUENTLY.THE.43S.COMPLEX	31	2.060	0.001
NONSENSE.MEDIATED.DECAY.NMD.ENHANCED.BY.THE.EXON.JUNCTION.COMPLEX.EJC.	64	2.059	0.001
NONSENSE.MEDIATED.DECAY.NMD.	64	2.045	0.001
RIBOSOMAL.SCANNING.AND.START.CODON.RECOGNITION	37	2.040	0.001
ACTIVATION.OF.THE.MRNA.UPON.BINDING.OF.THE.CAP.BINDING.COMPLEX.AND.EIFS.AND.SUBSEQUENT.BINDING.TO.43S	38	2.032	0.002
WP163.CYTOPLASMIC.RIBOSOMAL.PROTEINS	56	2.021	0.002
RESPIRATORY.ELECTRON.TRANSPORT.ATP.SYNTHESIS.BY.CHEMIOSMOTIC.COUPLING.AND.HEAT.PRODUCTION.BY.UNCOUPLING.PROTEINS.	82	1.947	0.005
WP1248.OXIDATIVE.PHOSPHORYLATION	47	1.929	0.007
CHEMOKINE.RECEPTORS.BIND.CHEMOKINES	40	1.925	0.007
THE.CITRIC.ACID.TCA.CYCLE.AND.RESPIRATORY.ELECTRON.TRANSPORT	120	1.903	0.009
KEGG_OXIDATIVE.PHOSPHORYLATION	109	1.890	0.010

WP295.ELECTRON.TRANSPORT.CHAIN	79	1.866	0.014
MITOCHONDRIAL.TRANSLATION.TERMINATION	81	1.773	0.041
INFLUENZA.VIRAL.RNA.TRANSCRIPTION.AND.REPLICATION	84	1.764	0.045

B

**Gene set**	**Size**	**NES**	**FDR q-val**
MITOTIC.PROMETAPHASE	104	-2.779	0.000
RESOLUTION.OF.SISTER.CHROMATID.COHESION	96	-2.759	0.000
KEGG_COMPLEMENT.AND.COAGULATION.CASCADES	76	-2.654	0.000
WP449.COMPLEMENT.AND.COAGULATION.CASCADES	61	-2.568	0.000
WP1.STATIN.PATHWAY	19	-2.483	0.000
FORMATION.OF.FIBRIN.CLOT.CLOTTING.CASCADE.	36	-2.397	0.000
INTRINSIC.PATHWAY	23	-2.278	0.000
LIPOPROTEIN.METABOLISM	27	-2.300	0.000
LIPID.DIGESTION.MOBILIZATION.AND.TRANSPORT	45	-2.340	0.000
COMMON.PATHWAY	17	-2.389	0.000
KINESINS	25	-2.199	0.001
BINDING.AND.UPTAKE.OF.LIGANDS.BY.SCAVENGER.RECEPTORS	36	-2.163	0.002
XENOBIOTICS	18	-2.118	0.005
WP190.CELL.CYCLE	84	-2.103	0.005
KEGG_RETINOL.METABOLISM	80	-2.105	0.005
MITOTIC.M.M.G1.PHASES	280	-2.084	0.005
LOSS.OF.PROTEINS.REQUIRED.FOR.INTERPHASE.MICROTUBULE.ORGANIZATION.FROM.THE.CENTROSOME	57	-2.076	0.006
WP460.BLOOD.CLOTTING.CASCADE	20	-2.064	0.006
RECRUITMENT.OF.MITOTIC.CENTROSOME.PROTEINS.AND.COMPLEXES	65	-2.046	0.007
LOSS.OF.NLP.FROM.MITOTIC.CENTROSOMES	57	-2.057	0.007
COMPLEMENT.CASCADE	31	-2.047	0.007
CELL.CYCLE.MITOTIC	414	-2.038	0.007
BIOC_INTRINSICPATHWAY	22	-2.028	0.008
WP200.COMPLEMENT.ACTIVATION.CLASSICAL.PATHWAY	16	-2.027	0.008
CELL.CYCLE	484	-2.018	0.009
REV.MEDIATED.NUCLEAR.EXPORT.OF.HIV.RNA	29	-2.008	0.009
CENTROSOME.MATURATION	65	-2.001	0.010
M.PHASE	247	-1.996	0.010
REGULATION.OF.GLUCOKINASE.BY.GLUCOKINASE.REGULATORY.PROTEIN	26	-1.985	0.010
TRANSPORT.OF.RIBONUCLEOPROTEINS.INTO.THE.HOST.NUCLEUS	28	-1.991	0.010
EXPORT.OF.VIRAL.RIBONUCLEOPROTEINS.FROM.NUCLEUS	30	-1.985	0.011
MITOTIC.METAPHASE.AND.ANAPHASE	166	-1.969	0.012
AMINO.ACID.AND.OLIGOPEPTIDE.SLC.TRANSPORTERS	49	-1.950	0.012
ERK.MAPK.TARGETS	21	-1.963	0.012

NUCLEAR.PORE.COMPLEX.NPC.DISASSEMBLY	29	-1.951	0.013
G2.M.TRANSITION	102	-1.951	0.013
WP317.GLYCOGEN.METABOLISM	34	-1.957	0.013
SEPARATION.OF.SISTER.CHROMATIDS	154	-1.952	0.013
MITOTIC.ANAPHASE	165	-1.940	0.013
INTERACTIONS.OF.REV.WITH.HOST.CELLULAR.PROTEINS	31	-1.935	0.014
DEPOSITION.OF.NEW.CENPA.CONTAINING.NUCLEOSOMES.AT.THE.CENTROMERE	36	-1.930	0.014
TRANSPORT.OF.MATURE.MRNAS.DERIVED.FROM.INTRONLESS.TRANSCRIPTS	32	-1.925	0.015
NEP.NS2.INTERACTS.WITH.THE.CELLULAR.EXPORT.MACHINERY	29	-1.921	0.015
NUCLEAR.ENVELOPE.BREAKDOWN	46	-1.909	0.016
NUCLEAR.IMPORT.OF.REV.PROTEIN	28	-1.914	0.016
FACTORS.INVOLVED.IN.MEGAKARYOCYTE.DEVELOPMENT.AND.PLATELET.PRODUCTION	116	-1.911	0.016
NUCLEOSOME.ASSEMBLY	36	-1.904	0.016
MITOTIC.G2.G2.M.PHASES	104	-1.895	0.017
PLATELET.DEGRANULATION	76	-1.889	0.018
TRANSPORT.OF.THE.SLBP.DEPENDANT.MATURE.MRNA	29	-1.883	0.018
INITIAL.TRIGGERING.OF.COMPLEMENT	17	-1.876	0.019
TRANSPORT.OF.MATURE.MRNA.DERIVED.FROM.AN.INTRONLESS.TRANSCRIPT	31	-1.868	0.020
PPARA_TARGETS	178	-1.866	0.021
RESPONSE.TO.ELEVATED.PLATELET.CYTOSOLIC.CA2.	81	-1.863	0.021
TRANSPORT.OF.THE.SLBP.INDEPENDENT.MATURE.MRNA	28	-1.837	0.027
FCERI.MEDIATED.CA.2.MOBILIZATION	25	-1.820	0.029
BUTYRATE.RESPONSE.FACTOR.1.BRF1.DESTABILIZES.MRNA	17	-1.821	0.030
UNFOLDED.PROTEIN.RESPONSE.UPR.	76	-1.815	0.030
TRANSPORT.TO.THE.GOLGI.AND.SUBSEQUENT.MODIFICATION	35	-1.822	0.030
IRE1ALPHA.ACTIVATES.CHAPERONES	46	-1.816	0.030
BIOC_ATRBRCAPATHWAY	19	-1.808	0.031
SYNTHESIS.OF.PIPS.AT.THE.GOLGI.MEMBRANE	17	-1.808	0.031
POST.TRANSLATIONAL.PROTEIN.MODIFICATION	244	-1.799	0.032
ANTIVIRAL.MECHANISM.BY.IFN.STIMULATED.GENES	58	-1.802	0.032
NUCLEAR.EVENTS.KINASE.AND.TRANSCRIPTION.FACTOR.ACTIVATION.	24	-1.800	0.032
CHYLOMICRON.MEDIATED.LIPID.TRANSPORT	16	-1.794	0.033
EXTRACELLULAR.MATRIX.ORGANIZATION	251	-1.785	0.035
KEGG_STEROID.HORMONE.BIOSYNTHESIS	80	-1.776	0.037
KEGG_PRION.DISEASES	35	-1.769	0.037
ISG15.ANTIVIRAL.MECHANISM	58	-1.771	0.038
AMINO.ACID.TRANSPORT.ACROSS.THE.PLASMA.MEMBRANE	31	-1.769	0.038
KEGG_PPAR.SIGNALING.PATHWAY	79	-1.764	0.038

REGULATION.OF.COMPLEMENT.CASCADE	19	-1.772	0.038
RORA.ACTIVATES.CIRCADIAN.GENE.EXPRESSION	24	-1.764	0.038
GLUCOSE.TRANSPORT	36	-1.759	0.039
PRE.NOTCH.PROCESSING.IN.GOLGI	18	-1.751	0.041
WP2316.PPAR.SIGNALING.PATHWAY	78	-1.753	0.041
KEGG_AMPK.SIGNALING.PATHWAY	126	-1.749	0.041
PHASE.1.FUNCTIONALIZATION.OF.COMPOUNDS	76	-1.739	0.044
REGULATION.OF.PLK1.ACTIVITY.AT.G2.M.TRANSITION	70	-1.735	0.044
O.GLYCOSYLATION.OF.TSR.DOMAIN.CONTAINING.PROTEINS	39	-1.736	0.045
VPR.MEDIATED.NUCLEAR.IMPORT.OF.PICS	28	-1.719	0.050
INTERACTIONS.OF.VPR.WITH.HOST.CELLULAR.PROTEINS	29	-1.715	0.050
REGULATION.OF.LIPID.METABOLISM.BY.PEROXISOME.PROLIFERATOR.ACTIVATED.RECEPTOR.ALPHA.PPARALPHA.	114	-1.716	0.051
PI.METABOLISM	50	-1.709	0.052
