# Supplementary material for: Lifestyle and Horizontal Gene Transfer-Mediated Evolution of Mucispirillum schaedleri, a Core Member of the Murine Gut Microbiota
Source: mSystems. 2017 Jan 31;2(1):e00171-16. doi: 10.1128/mSystems.00171-16 (PMC5285517; doi:10.1128/mSystems.00171-16)
Supplement: TABLE S5 [file sys001172082st10.pdf]

Table S5. Predicted factors responsible for the regulation of the differentially expressed genes in mice colonized with *M. schaedleri* MCS. All mice had an ASF[4] microbiota as a background (n=6 per group). The differential expression levels of the listed target genes were used to predict whether an upstream regulator was activated or inhibited. The differential expression of the predicted upstream regulator is shown (Exp. Fold Change). Activation z-score and the associated p-value are based on a null model of no association based on random permutation of expression levels.

Upstream Regulator	Exp. Fold Change	Molecule Type	Predicted State	Activation z-score	p-value	Target genes in dataset
RELA	-1.075	transcription regulator	Activated	2.581	2.78E-02	ANKRD1,BNIP3L,CCL11,ELN,FOS,Madcam1,TLR2,TRAF1
NFkB (complex)		complex	Activated	2.566	2.17E-04	ANKRD1,CCL11,CLU,Cxcl9,DBP,FOS,FST,IFNA4,Madcam1,NR1D1,PIM3,SLC22A4,TFRC,TLR2,TRAF1,VPREB1
tamoxifen		chemical drug	Activated	2.387	1.15E-02	CLU,CYP2E1,FASN,FOS,OXTR,SCD,Scd2
poly rI:rC-RNA		biologic drug	Activated	2.285	2.65E-03	CCL11,Cxcl9,FOS,IFNA4,Ins1,LPL,NFIL3,PIM3,TLR2,TRAF1,TSHR
PPARD	1.030	ligand-dependent nuclear receptor	Activated	2.236	3.14E-02	FASN,LPL,PER3,SCD,Tlr12
arachidonic acid		chemical - endogenous mammalian	Activated	2.227	1.73E-03	CLU,FASN,FOS,SCD,THRSP
CYB5R4	1.056	enzyme	Activated	2.219	1.05E-06	FASN,Ins1,LPL,SCD,Scd2
beta-carotene		chemical - endogenous mammalian	Activated	2.219	2.58E-05	LPL,ME1,NPR3,Orm1 (includes others),Scd2
hyaluronic acid		chemical - endogenous mammalian	Activated	2.183	3.52E-03	Cxcl9,FOS,FST,HMMR,TLR2
HOXA10	1.029	transcription regulator	Activated	2.111	1.28E-06	ALAS2,CA3,CYP2E1,DBP,FST,GSTA3,HLF,ME1,SCD,Scd2,THRSP
POR	-1.021	enzyme	Activated	2	7.99E-03	EREG,GSTA3,HLF,LPL,SCD,Scd2
EGF	1.048	growth factor	Inhibited	-2.016	2.47E-04	CLU,EREG,FASN,FOS,FST,Ins1,KRT16,MNX1,PBK,REN,SCD,Scd2,SERPINA1,ST3GAL3

rosiglitazone		chemical drug	Inhibited	-2.051	2.05E-04	CCL11,Cxcl9,FASN,FOS,HP,KLB,LPL,NR1D1,PC,RARB,REN,SCD,TSHR
PD98059		chemical - kinase inhibitor	Inhibited	-2.18	2.05E-05	ANKRD1,CCL11,CLU,DSG1,ELN,FASN,FOS,Ins1,LPL,PC,REN,SEC14L2,SERPINA1,TF,TLR2,TNNI1,TRAF1
THRB	1.004	ligand-dependent nuclear receptor	Inhibited	-2.216	9.30E-03	FASN,FOS,LPL,ME1,THRSP,TSHR
IGF1	-1.072	growth factor	Inhibited	-2.328	3.14E-05	CLU,EDN2,ELN,FASN,FOS,HBB,Ins1,LPL,PBK,RARB,SCD,TF,THRSP,TSHR
Insulin		group	Inhibited	-2.358	3.90E-04	CA3,FASN,FOS,LPL,ME1,NFIL3,SCD,Scd2,THRSP,TLR2,TSHR
RXRA	1.073	ligand-dependent nuclear receptor	Inhibited	-2.369	8.75E-04	FASN,FOS,LPL,NR1D1,Orm1 (includes others),PC,RARB,REN,SCD
